# Supplementary material for: Unique reactivity of nanoporous cellulosic materials mediated by surface-confined water
Source: Nat Commun. 2021 May 4;12:2513. doi: 10.1038/s41467-021-22682-3 (PMC8097012; doi:10.1038/s41467-021-22682-3)
Supplement: Supplementary file 1 — Supplementary Information [file 41467_2021_22682_MOESM1_ESM.pdf]

Supplementary Information for

Unique Reactivity of Nanoporous Cellulosic Materials Mediated by Surface-Confined Water

Marco Beaumont*, Paul Jusner, Notburga Gierlinger, Alistair W. T. King, Antje Potthast, Orlando J. Rojas, Thomas Rosenau*

Correspondence to: thomas.rosenau@boku.ac.at, marcobeaumont1@gmail.com

This PDF file includes:

Supplementary Text
Supplementary Methods
Supplementary Section 1
Supplementary Figures 1 to 36
Supplementary Tables 1 to 3
Supplementary References
Data S1: Optimised geometries (DFT level)

Supplementary Text

Supplementary Table 1: Overview of native cellulose fibre specifications.

Properties	Type of fibre	Beech sulfite dissolving pulp
	Glucan content	92.7 (1, *p. 1036*)
	Hemicellulose content / %	3.8 [a]
	Carbonyl content / µmol/g	18.8 (1, *p. 1036*)
	Weight-averaged degree of polymerization	1790 (1, *p. 1036*)
	Average pore diameter / nm	5.1 (1, *p. 1053*)
	Crystallinity / %	48 [a]
	Elementary fibril diameter / nm	3.4 [a]
	Specific surface area / m^2/g	124 [b]
	Specific pore surface / m^2/g	235 (1, *p. 1053*)
Reactivity	Superficial hydroxyl groups / mmol/mmol[f]	0.75 [c], 0.74 [d]
Water	Fibre saturation point / mL/g	0.50 (1, *p. 1053*)
	Water retention value / mL/g	0.73 (1, *p. 1053*)
	Pore volume / mL/g	0.60 (1, *p. 1053*)
	Equilibrated moisture content (50% RH) / wt%	7 [e]
	Equilibrated moisture content (95% RH) / wt%	17 [b]

[a] Solid-state NMR analysis in **Supplementary Figure 9A**. [b] From dynamic vapour sorption in **Supplementary Figure 10**. [c] Theoretical accessible hydroxyl groups based on 24-chain model in **Supplementary Figure 11.** [d] Surface cellulose hydroxyl groups were calculated from the crystallite size according to Okita *et al.*(2). [e] Average value from at least four moisture content measurements of the fibre. [f] Molar amount of accessible hydroxyl groups per mmol of glucose unit.

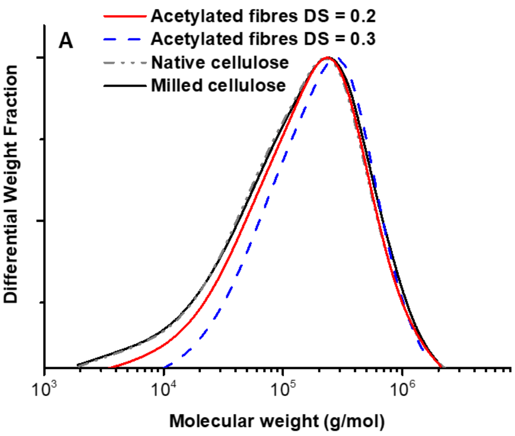

B

Sample	NMR crystallinity
Native cellulose	48%
Milled cellulose	47%
Acetylated fibres DS = 0.2	46%
Acetylated fibres DS = 0.3	48%

Supplementary Figure 1: Molar mass distribution of the native cellulose, the cellulose after milling and acetylated cellulose fibre samples (A). Comparison of the NMR crystallinity indices of these samples (B), extracted from their respective solid-state NMR spectra (**Supplementary Figures 12-15**) and peak deconvolution (**Supplementary Figure 9**) according to Wickholm *et al.(3*). The results show that the physical structure of the fibre was not affected under the used conditions. The shift in the molar mass distribution in case of acetylated fibres is due to the mass gain from the acetyl groups.

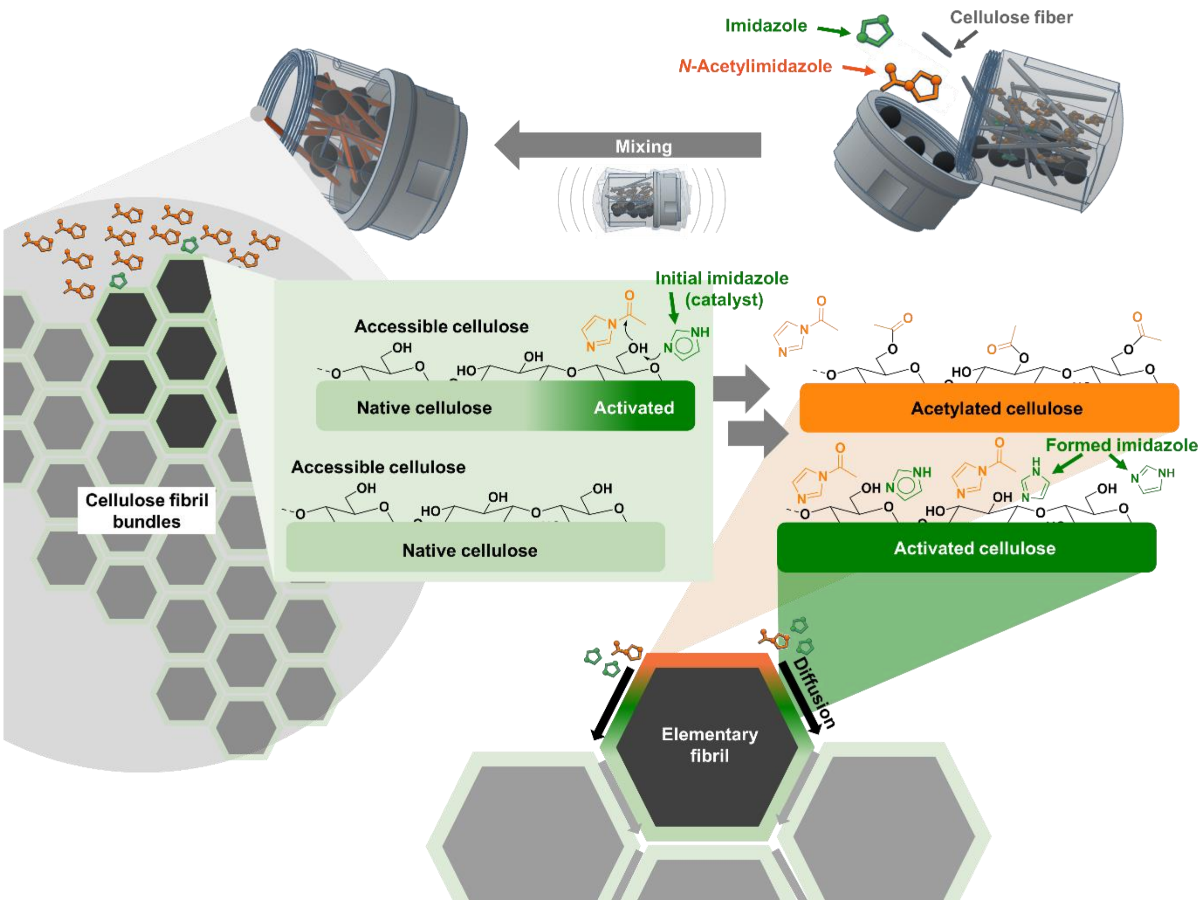

Supplementary Figure 2: Scheme of the solid-state acetylation reaction of cellulose fibres (the accessible elementary fibril, i.e. nanofibre, surface is covered with a hydration layer). The reaction is initiated by fast and efficient solid-state mixing of cellulose and reactants in a ball mill. The reaction progresses from the outer elementary fibrils located at the fibre surface, where the reactants are nanosolvated in the hydration layer. The catalytic imidazole activates the accessible cellulose hydroxyl groups and facilitates their acetylation. The reaction is autocatalytic and the formed imidazole (through acetyl transfer from *N*-acetylimidazole) additionally speeds up the reaction. The reactants diffuse further into the fibre along the elementary fibrils' hydration layer. Diffusion of *N*-acetylimidazole is expected to be the limiting factor, as it is significantly slower than the diffusion of imidazole because of the lower solubility of *N*-acetylimidazole in water (and hence also in the cellulose hydration layer).

Supplementary Table 2: Solid-state acetylation of cellulose and influence of the moisture content on the degree of substitution. EMC = equilibrated moisture content at 50% relative humidity and 20 °C was approx. 7 wt%. Mean values and standard deviations were calculated from three measurements of two samples each (except for * marked samples, three measurement of one sample each). Standard deviations are listed in parentheses.

Equivalents of *N*-acetylimidazole	Moisture content	Degree of substitution	Reaction efficiency[b] / %
0.3	EMC (7 wt%)	0.23(0.01)	80(4)
0.3	dry	0.25(0.01)*	87(5)*
0.3	DMSO[a]	0.24(0.00)*	85(0)*
1.0	EMC (7 wt%)	0.34(0.02)	35(2)
1.5	EMC (7 wt%)	0.49(0.02)	34(1)
1.5	dry	0.38(0.01)	26(1)
1.5	20 wt%	0.27(0.02)	19(2)
1.5	30 wt%	0.20(0.04)	14(3)

[a] Fibre was treated with DMSO using the same volume as water in the EMC sample. [b] The reaction efficiency was calculated from the ratio of the degree of substitution and the molar amount of *N*-acetylimidazole (95% purity).

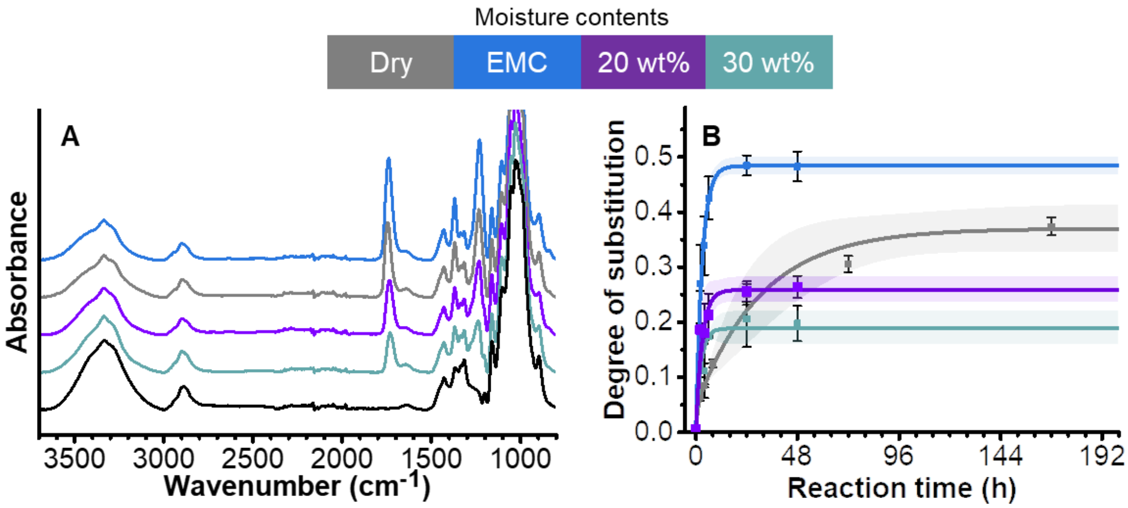

Supplementary Figure 3: A: Infrared spectra of the acetylated fibres from the reaction with 1.5 eq. *N*-acetylimidazole at different moisture contents from 0 wt% (dry) to 30 wt%. EMC is the equilibrated moisture content at a relative humidity of 50%, approx. 7 wt%. B: Dependence of the degree of substitution on the reaction time. The dry sample was fitted with an ExpAssoc fit and the remaining data with exponential fits (R-Square > 0.99). 95% confidence bands of the respective fits are shown in shaded colour. Error bars represent two standard deviations from the mean values (three measurement of one sample each; except for t = 0 h (five measurements of one sample), and EMC t = 2 h, 4 h and 6 h (three measurements of two samples each)).

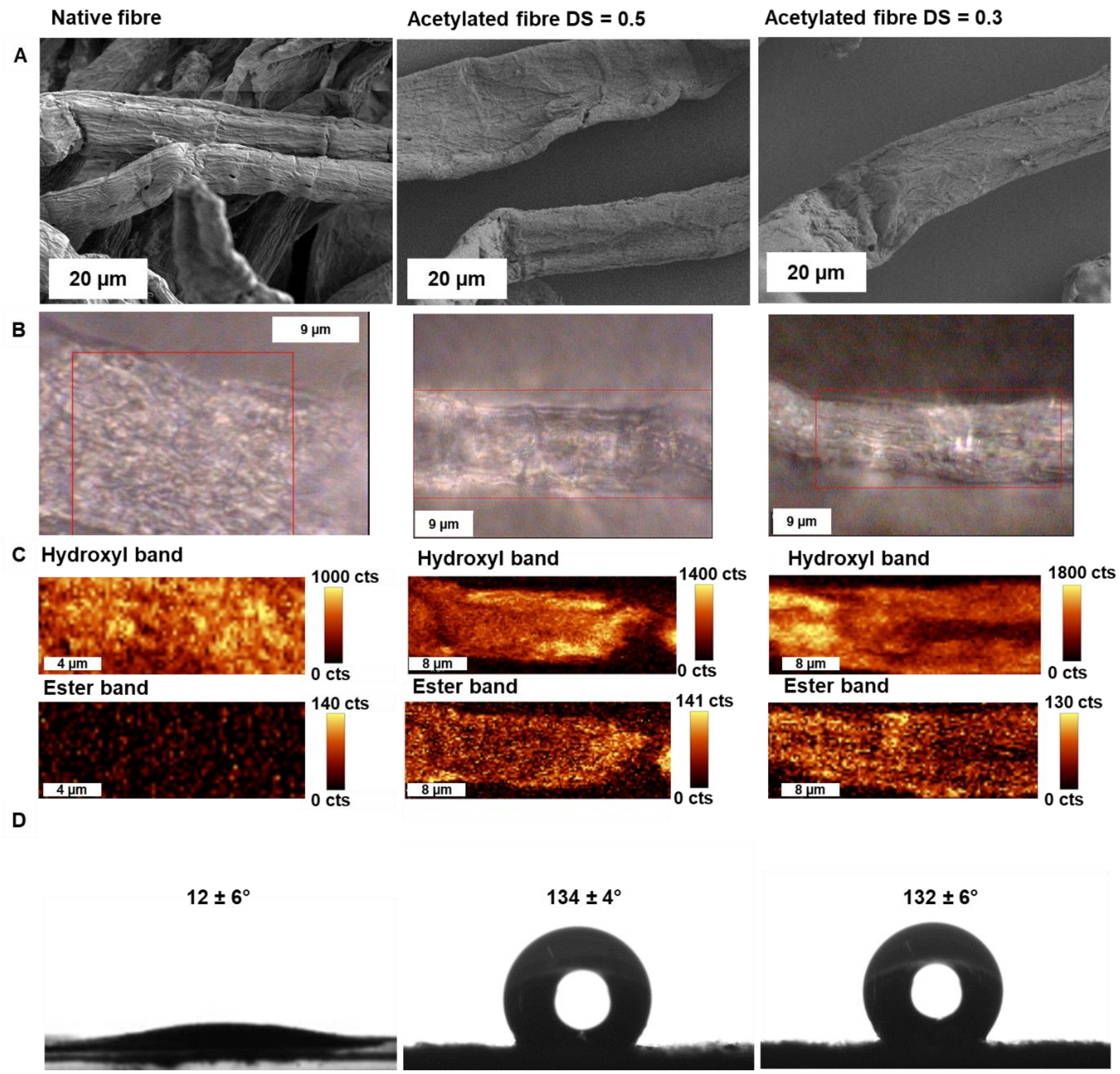

Supplementary Figure 4: Characterization of acetylated fibres with different degree of substitution (DS) in comparison to the native starting material. Scanning electron microscopy images of fibres (A row), light microscopy images of the scanned region for Raman analyses (B row) and Raman chemical imaging visualizing the surface distribution of hydroxyl (at 3400 cm^{-1}) and ester bands (at 1730 cm^{-1}) (C row). Raman signal intensities in counts (cts), measured with a charge-coupled device (CCD), are represented by the colour scales. Water droplet on the respective fibre and corresponding water contact angles are shown in D row. Although the DS of the acetylated varies significantly Raman imaging and water contact angle of the sample gave similar results, proving a homogeneous density of acetyl groups on the outer surface of the cellulose fibres. Mean values and standard deviations of contact angle values were calculated from four measurements of one sample.

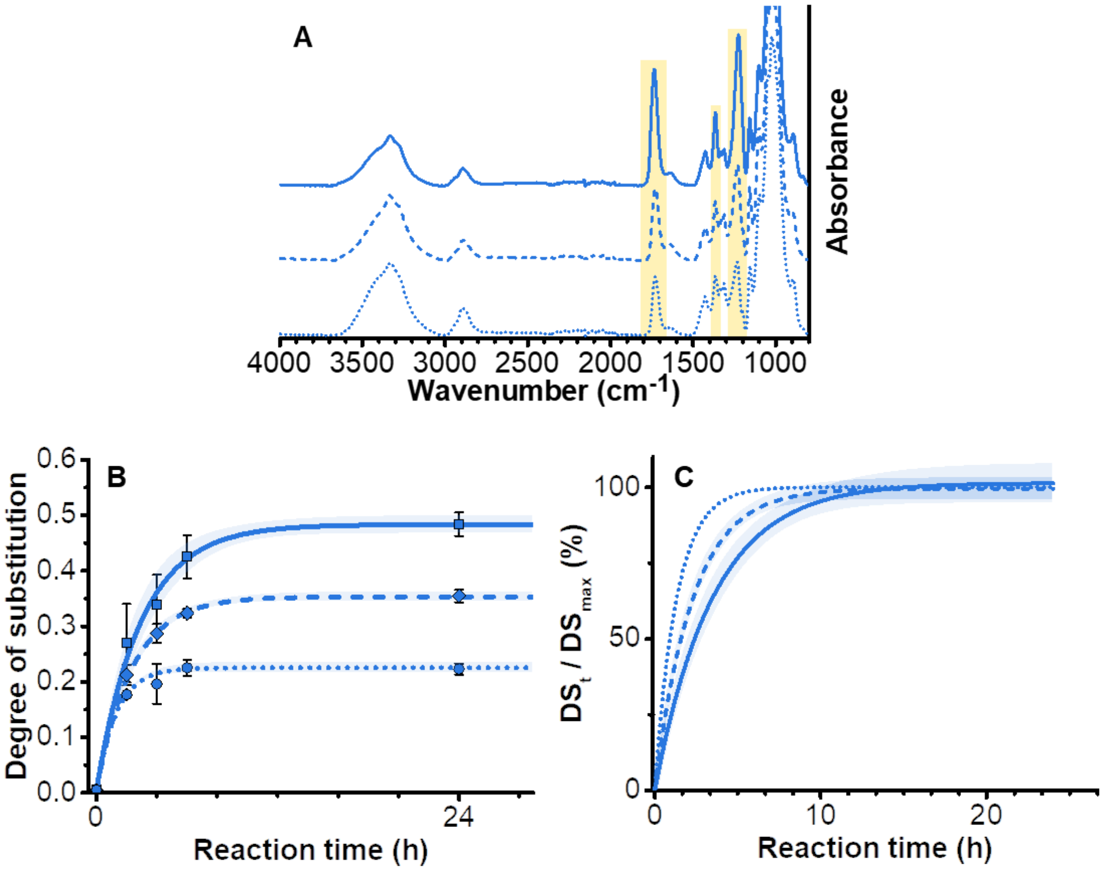

Supplementary Figure 5: IR spectra of acetylated fibres and resulting reaction kinetics. A: Reaction with 0.3 eq (equivalents based on cellulose monomer unit) (dotted line), 1.0 eq. (dashed line) and 1.5 eq. of *N*-acetylimidazole (solid line). B: Dependence of the degree of substitution on the reaction time, C: Progress of the reaction visualised as reaction time vs. the ratio of DS and final maximal DS (DS_{max}) (C). All scatter plots were fitted with an exponential fit (R-Square > 0.99) and their 95% confidence bands are shown in shaded colour. Error bars represent two standard deviations from the mean values (three measurement of one sample each; except for t = 0 h (five measurements of one sample), and 1.5 Eq t = 2 h, 4 h and 6 h (three measurements of two samples each)).

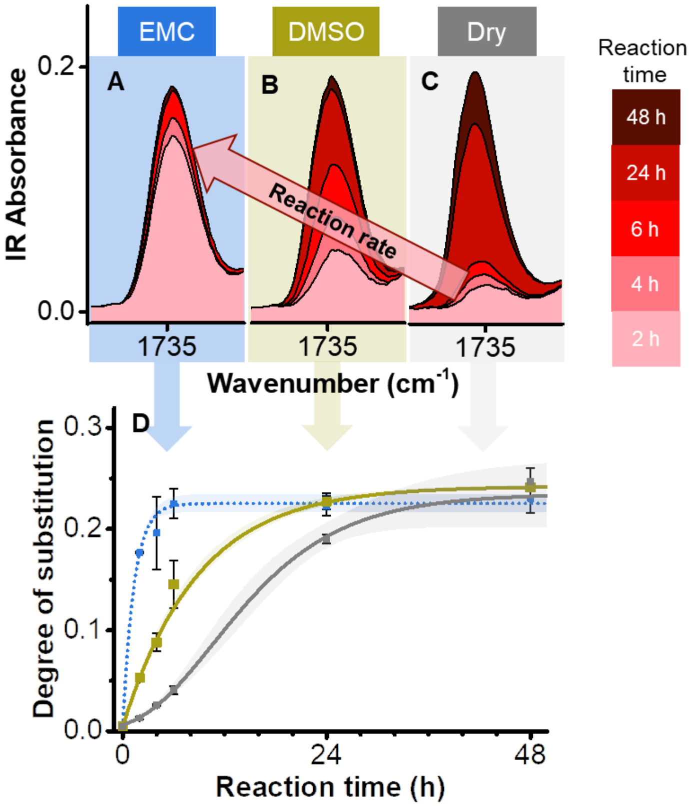

Supplementary Figure 6: Comparison of acetylation of fibres with 0.3 eq. of *N*-acetylimidazole under different conditions: fibres at EMC (equilibrated moisture content at 50% relative humidity) (A, blue squares and dotted line), fibres treated with DMSO (same volume as water in fibre at EMC) (B, golden squares and line) and dried fibres (0 wt% moisture content, C, grey squares and line). Increase of the carbonyl band during the reaction (A-C) and extracted kinetic data (D). Blue and golden lines were fitted with exponential fits (R-square > 0.9999) and grey line was fitted with sigmoidal SGompertz function fit (R-square > 0.99). 95% confidence bands of the respective fits are shown in shaded colour. Error bars represent two standard deviations from the mean values (three measurement of one sample each; except for t = 0 h (five measurements of one sample)).

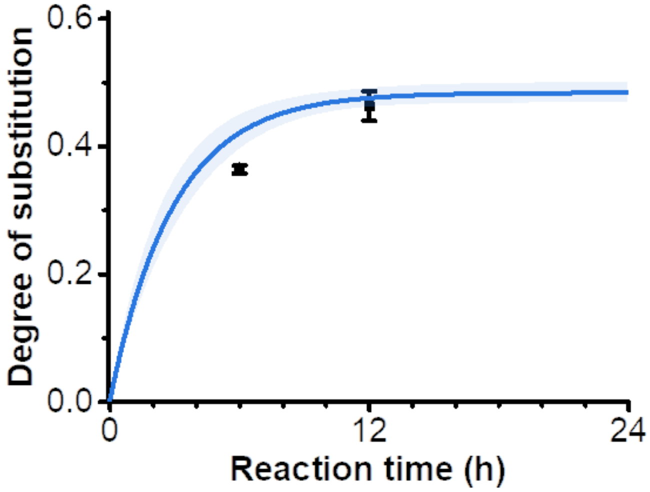

Supplementary Figure 7: Comparison of acetylation of non-pre-treated cellulose fibres (black squares) with acetylation of pre-treated cellulose fibres (blue line). Both reactions were conducted at 1.5 equivalents. All reactions were performed according to standard conditions using fibres at EMC (see Methods in main text). The pre-treatment is not mandatory and only increased initially the reaction rate; after longer reaction time degree of substitution of pre-treated and non-treated fibres were indistinguishable. Exponential fit (R-Square > 0.99) of reaction kinetics, 95% confidence bands of the fit is shown in shaded blue colour. Error bars represent two standard deviations from the mean values (three measurements of one sample each).

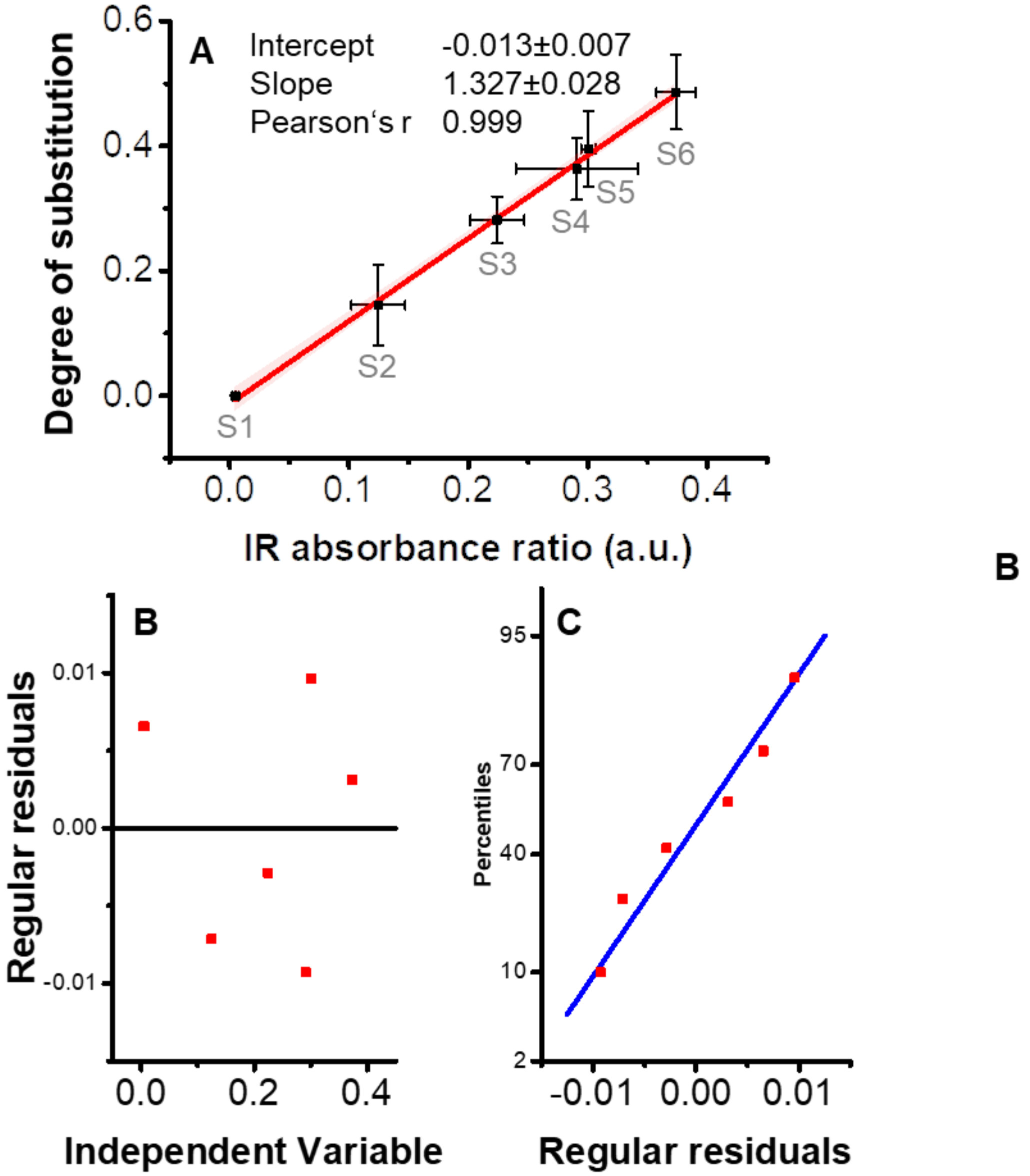

Supplementary Figure 8: Calibration curve based on infrared spectroscopy (in **Supplementary Figure 16**) and degree of substitution determined by the titration method. Residual vs. independent plot (B) and normal probability plot of residuals (C) of the linear fit. The IR carbonyl absorbance was obtained from the maximum height of the IR carbonyl band at approx. 1730 cm^{-1}. Error bars represent two standard deviations from the mean values (three measurements of one sample each; except for, S1 and S5 IR absorbances five measurements of one sample each, S2 degree of substitution duplicate measurement of one sample).

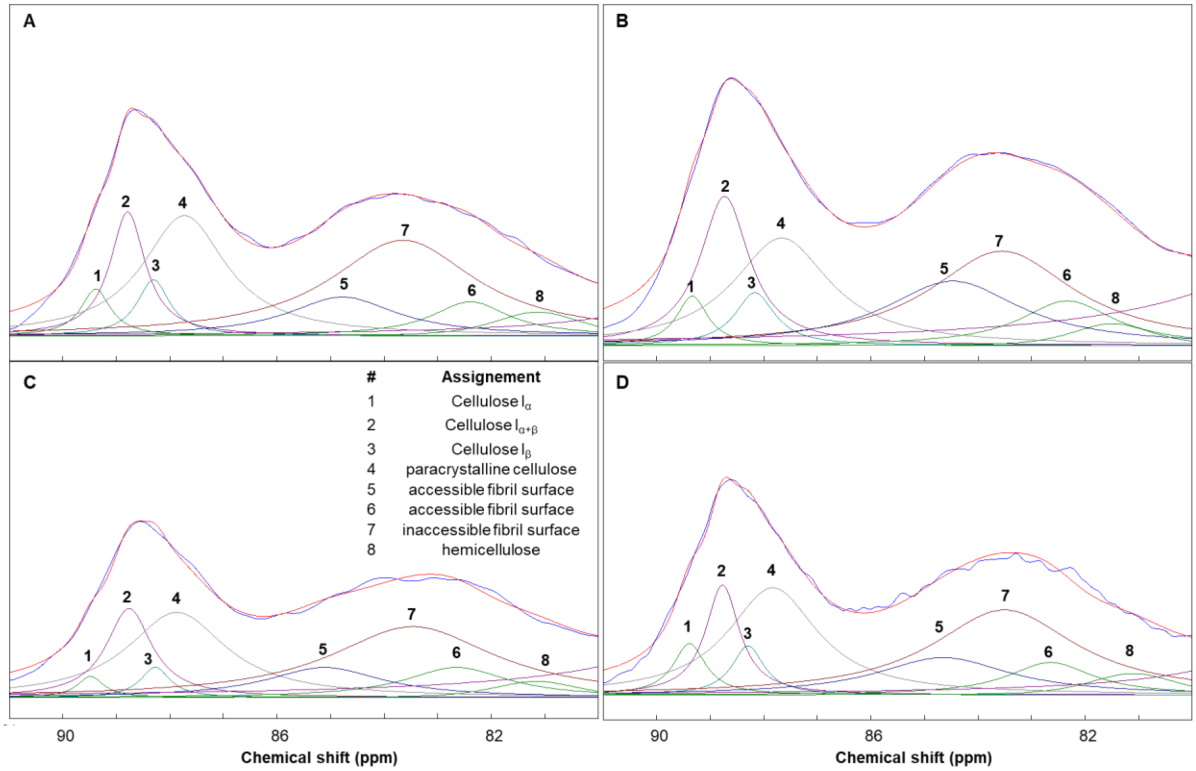

Supplementary Figure 9: Deconvolution of solid-state ^{13}C NMR spectra of the native cellulose fibre (A), the milled fibre (B) and acetylated fibres (DS =0.2 (C) and DS = 0.3 (D)) for determination of sample crystallinity according to Wickholm *et al*.(3). The elementary fibril diameter was calculated for the native cellulose sample A. Full spectra are shown in **Supplementary Figures 12-15**.

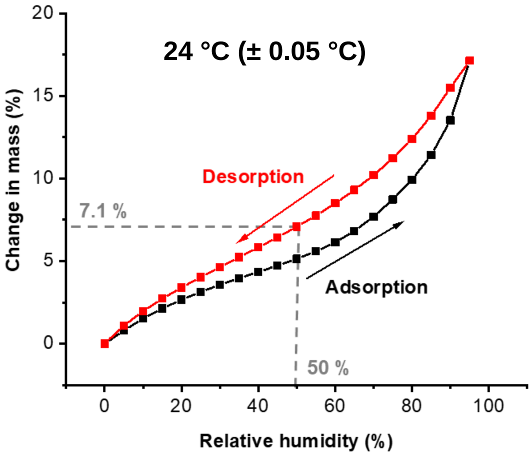

Supplementary Figure 10: Water vapour sorption of the native fibre measured by dynamic vapour sorption at 24 °C. The equilibrated moisture content at 50% relative humidity is indicated by a dashed line.

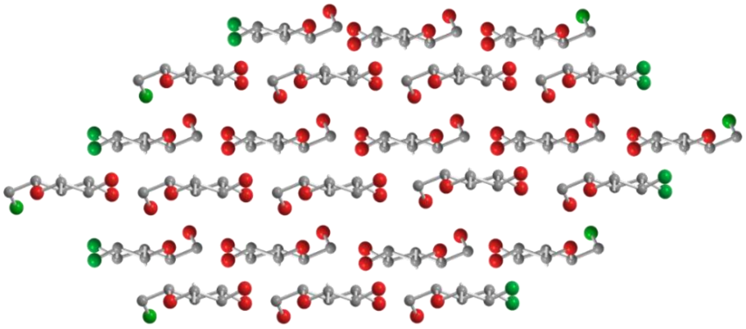

Supplementary Figure 11: Hexagonal 24-chain model of the cellulose elementary fibril (*4–7*), C6-OH, C2-OH and C3-OH are coloured in red or green. Non-accessible groups are in red and accessible surface hydroxyl groups are in green colour. The theoretical number of accessible hydroxyl groups expressed in equivalents based on the cellulose monomer unit is 0.75.

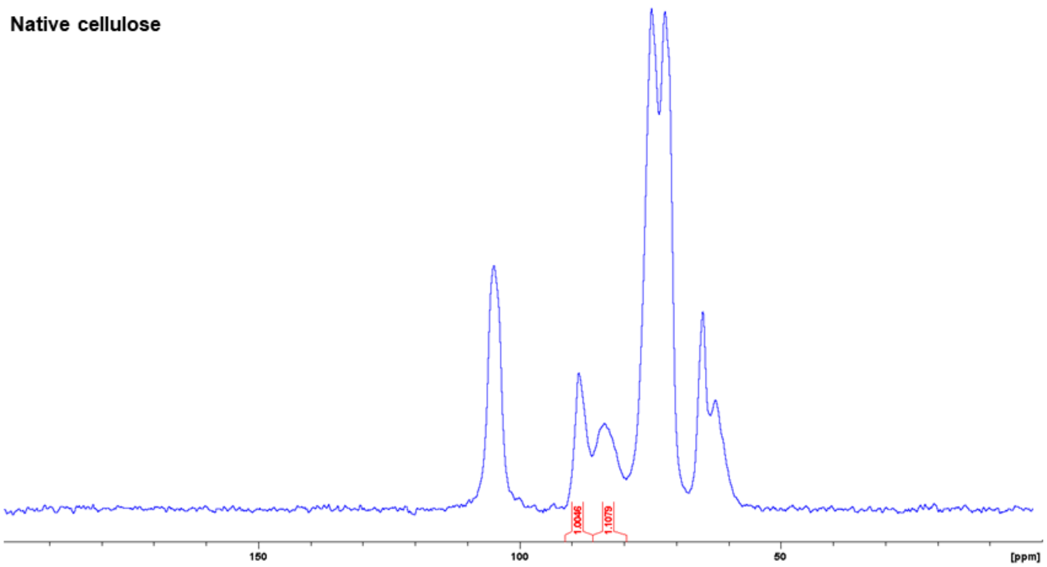

Supplementary Figure 12: Solid-state NMR spectrum of native cellulose fibres.

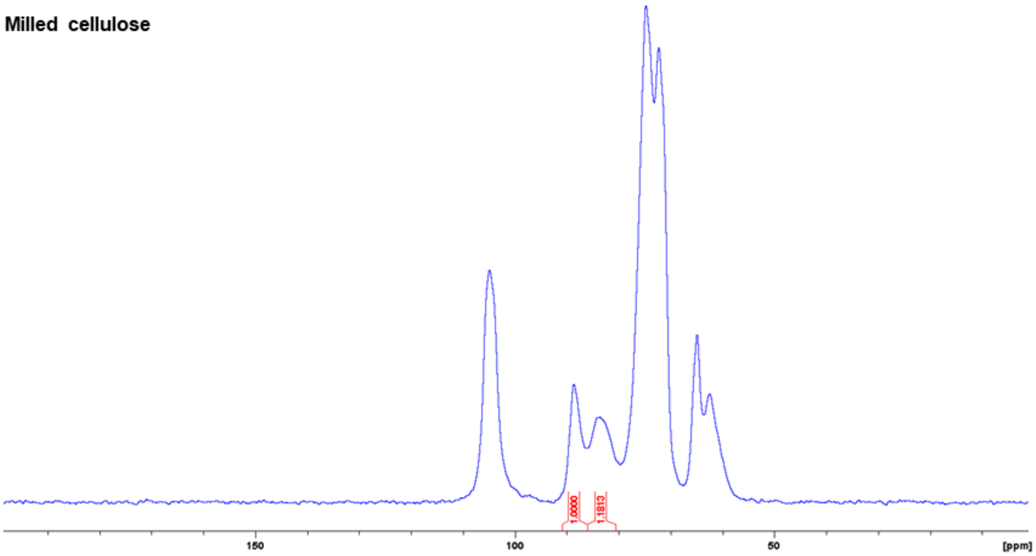

Supplementary Figure 13: Solid-state NMR spectrum of milled cellulose fibres.

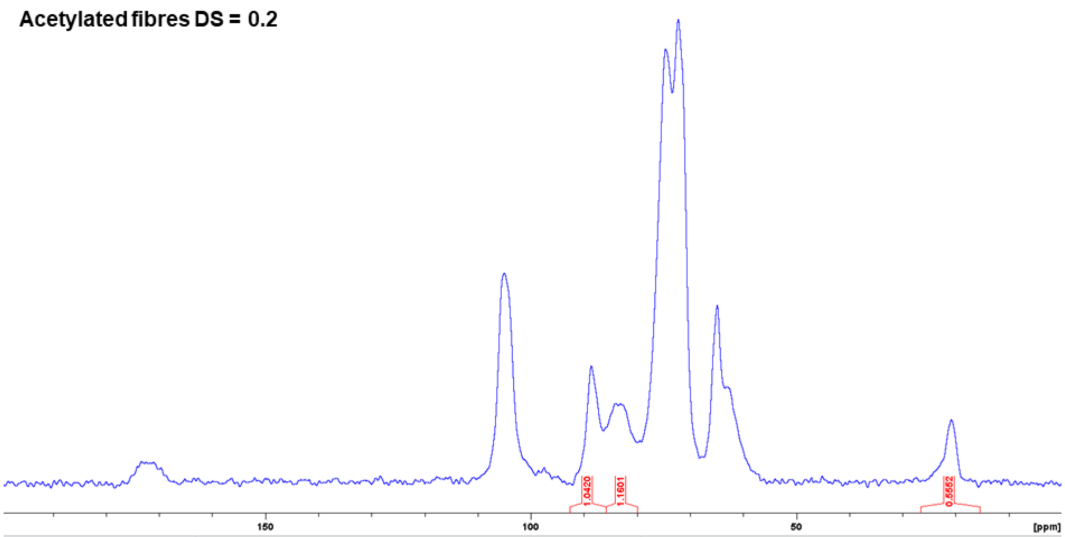

Supplementary Figure 14: Solid-state NMR spectrum of acetylated fibres with a degree of substitution of 0.2.

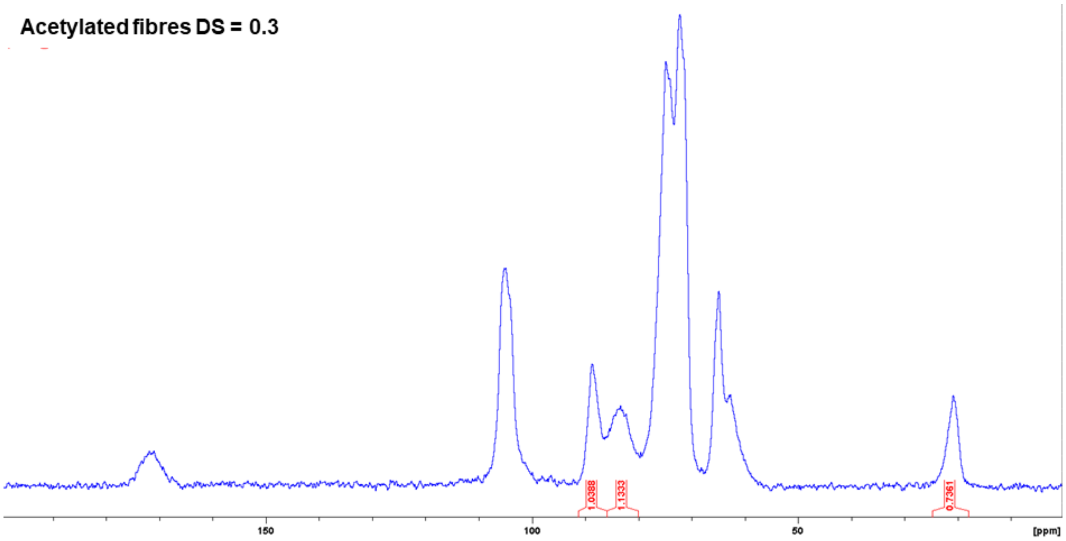

Supplementary Figure 15: Solid-state NMR spectrum of acetylated fibres with a degree of substitution of 0.3.

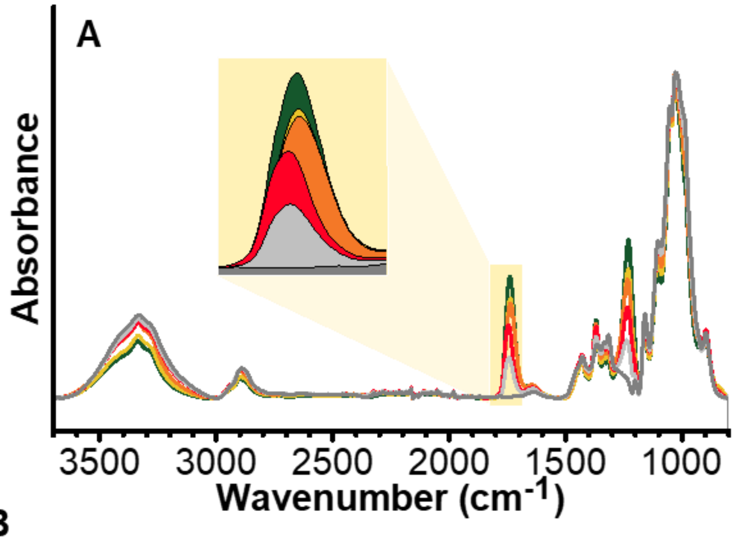

B

#	IR C=O absorbance	Degree of substitution
1	0.00(0.00)	-
2	0.12(0.02)	0.15(0.06)
3	0.22(0.02)	0.28(0.04)
4	0.29(0.05)	0.36(0.01)
5	0.30(0.01)	0.40(0.06)
6	0.37(0.02)	0.49(0.06)

Supplementary Figure 16: A: Infrared spectra of acetylated fibre samples with different degrees of acetylation. B: IR C=O absorbance extracted from the IR spectra. These values were used to build the calibration curve plot in Supplementary Figure 8. Mean values and standard deviations were calculated from three measurements of one sample each; except for, Samples #1 and #5 IR absorbances five measurements of one sample each, Sample #2 degree of substitution duplicate measurements of one sample).

Supplementary Methods

Fourier transformed infrared spectroscopy (IR)

IR spectra were recorded in the range between 4000 cm^{-1} and 650 cm^{-1} with 4 scans per measurement at a resolution of 4 cm^{-1}, using a PerkinElmer Frontier IR single-range spectrometer (PerkinElmer Inc., USA) in attenuated total reflection (ATR) mode. The spectrometer was equipped with a ZnSe ATR crystal and a $LiTaO_3$ detector. All samples were dried at 105 °C to constant weight and equilibrated at room temperature and 50% relative humidity before measurement. The spectra were processed with the software Spectragryph (version 1.2.11, Dr. Friedrich Menges, Germany) by an adaptive base-line correction with a coarseness factor of 20 and normalization of the highest band (cellulose C-O stretching band at approx. 1029 cm^{-1}) to an absorbance of 1. The shown IR spectra are averaged spectra from triplicate measurements. The height of the carbonyl bands is defined as highest value in the range of 1745 cm^{-1} to 1730 cm^{-1} wavenumbers.

Solid-state nuclear magnetic resonance spectroscopy (NMR)

Solid state NMR experiments were measured on a Bruker Avance III HD 400 spectrometer (resonance frequency of ^{1}H of 400.13 MHz, and ^{13}C of 100.61 MHz, respectively), equipped with a 4 mm dual broadband CP-MAS probe. ^{13}C spectra were obtained by using the TOSS (total sideband suppression) sequence at ambient temperature with a spinning rate of 5000 Hz. The NMR experiment was conducted with a cross-polarization (CP) contact time of 2 ms, a recycle delay of 2 s, a SPINAL-64 ^{1}H decoupling and an acquisition time of 49 ms. The spectral width was set to 250 ppm. Chemical shifts were referenced externally against the carbonyl signal of glycine at $\delta = 176.03$ ppm. The acquired FIDs were apodised with an exponential function (lb = 11 Hz) prior to Fourier transformation. All materials for solid-state NMR were oven-dried at 105°C to remove excess of water and equilibrated at room temperature to constant weight before measurement. For standard data processing the software Bruker TopSpin 3.5 was used.

The crystallinity were determined by deconvolution of the crystalline and amorphous part of C4(*3*). This method was used to estimate the effect of reaction conditions on the crystallinity of the samples (**Supplementary Figures 13-16**).

Peak fitting of native cellulose solid-state NMR spectrum was done according to Wickholm *et al*.(*3*) using the program Dmfit (8) (**Supplementary Figure 12A**) and the elementary fibril diameter was calculated based on the work of Newman (*9*) using the lateral cellulose chain spacings calculated from the 24 chain model (5). The resulting data from the fittings was used further to calculate the amount of hemicellulose in the sample, as well as the crystallinity in **Supplementary Table 1**.

Determination of degree of substitution (DS) via titration

The samples were first dried at 105 °C to constant weight. Then 50 mg were accurately weighed in a 50 mL vessel. To every sample 5 mL ethanol (denaturated >96%), 2.5 mL DI water and 2.5 mL 1 M sodium hydroxide solution were added. The closed vessels containing the samples were shaken for 72 h. 20 mL of DI water and 1 mL of saturated NaCl solution were added and the samples were titrated with a 0.5 M hydrochloric acid solution using a Metrohm 877 Titrino plus titrator (Metrohm AG, Switzerland). The volume of hydrochloric acid solution consumed was determined at a fixed point of pH = 7. The results of the acetylated samples were compared to titration of a blank sample consisting of native cellulose.

These results were used to establish a calibration curve in combination with the carbonyl band absorbance from IR spectra (**Supplementary Figure 8**), allowing follow-up DS determination by simple IR measurement. Kinetics plots (reaction time vs. degree of substitution) were fitted with Origin Pro 2016 (OrigianLab Corporation, USA).

Raman microscopy

Raman imaging of native and acetylated cellulose fibres was done with a confocal Raman microscope (CRM) (Alpha300RA, WITec GmbH, Germany) equipped with a linear polarised (0°) coherent compass sapphire VIS laser (λ(ex) = 532 nm, laser power = 40 mW, WITec GmbH, Germany). The laser light was focused through a 100x oil immersion objective (numerical aperture = 1.4, coverslip corrected 0.17 mm) (Carl Zeiss, Germany) onto the fibres and the backscattered Raman signal directed through an optic multifibre (50 μm diameter) to a spectrometer (UHTS 300 WITec, Germany) (600 $g \cdot mm^{-1}$ grating) and detected by the CCD camera (Andor DU401 BV, Belfast, North Ireland). On selected areas (e.g. 50 μm x 20 μm) along the fibres every 0.3 μm a full wavenumber range (100 - 3800 cm^{-1}) Raman spectrum was acquired with an integration time of 0.13 s. The Control FOUR (WITec GmbH, Germany) acquisition software was used for the Raman measurements set up and Project FOUR (WITec GmbH, Germany) to reconstruct Raman images based on the integral band of the ester group at 1734 cm^{-1} and the hydroxyl groups at 3400 cm^{-1}.

Contact angle measurement

Cellulose samples were prepared on filter paper for contact angle measurements. 100 mg of every sample were dispersed in 20 mL of a 1:1 mixture DI water with ethanol and stirred overnight. The suspensions were treated with an ULTRA-TURRAX T8 (IKA-Werke GmbH & CO. KG, Germany) for 5 min at 9000 min^{-1} and vortexed. The suspensions were filtered under vacuum through a Büchner funnel to produce a dense filter cake on a Whatman Grade 1 filter paper. The filter cake was washed with acetone by filtration and dried at ambient conditions overnight to obtain a filter paper coated with the respective cellulose sample.

Finally, the coated filter was pressed at 90 °C for 9 min at a pressure of 1 bar to obtain a smooth and comparable surface. The contact angle measurements were carried out on a Drop Shape Analyzer DSA30 (KRÜSS Optronic, Germany) operated by the KRÜSS ADVANCE 1.5.1.0 software. The volume of every water drop was 2 μL at a dosage speed of 0.5 mL/min.

The contact angles were measured at 10 frames per second for 1 s and four measurements were performed for each sample. The curve fitting for the contact angle calculation was done by using a Young-Laplace function. Data analysis was performed with the KRÜSS ADVANCE 1.5.1.0 software.

Gel permeation chromatography (GPC)

For the GPC analysis approximately 15 mg of dried pulp samples were dispersed in 250 mL DI water and treated for approximately 20 s in a blender. The samples were filtered and washed with ethanol. Each sample was transferred in a 4 mL vial, 4 mL of *N,N*-dimethylacetamide (DMAc) was added and the suspension was shaken overnight. Afterwards, the excess of DMAc was removed by filtration and 2 mL of DMAc/LiCl (9%, w/v) were added and shaken at room temperature until complete dissolution of the cellulose sample. Finally, 0.3 mL of the sample were diluted with 0.9 mL DMAc and filtered through a 0.45 µm grid syringe filter.

The GPC measurements were done with a multiple-angle laser light scattering (MALLS) detector with an argon ion laser (λ = 488 nm) (Wyatt Dawn DSP, Wyatt Inc. Santa Barbara, USA) and a refractive index (RI) detector (Shodex RI-71, Showa Denko K.K., Japan). Of every sample 100 µL were injected with an Agilent HP series 1100 autosampler (Agilent, Waldbronn, Germany). A Bio-Inert 1260 Infinity II (Agilent, Waldbronn, Germany) was used and four serial GPC columns (Agilent PLgel Mixed ALS, 20 µm, 300 mm x 7.5 mm) were part of the system. The eluent was DMAc/LiCl (0.9%, w/v) at a flow rate of 1 mL/min and the run time was 45 min. The data was evaluated with Astra 4.7 software.

Dynamic vapor sorption

Water vapor sorption was measured at 24 °C (± 0.05 °C) with a Dynamic Vapor Sorption equipment (DVS Advantage ET, Surface Measurement Systems Ltd., United Kingdom). The weight of the sample was monitored with a microbalance whilst a constant flow of nitrogen of predetermined water content was used to control the RH within the sample chamber. The sorption–desorption isotherm cycles were collected sequentially for the same sample and the sample equilibrium moisture content (EMC) was recorded after the mass change rate stayed below 0.005 %/min continuously over a 10 min time-period. The measurement was conducted as one-cycle step process, in which the target humidity was varied from 0% to 95% relative humidity in 5% increments.

Solution-state NMR Supplementary Data

NMR Sample Preparation

To prepare the samples for NMR analysis, typically 50 mg of dried sample is added to a sealable sample vial and made up to 1 g, by addition of stock [P_{4444}][OAc]:DMSO-d_6 (20:80 wt%) electrolyte solution*(10,11)*. The samples were magnetically stirred at RT until they go clear. This typically was over a 1 h period. If the samples did not go clear during that period, the temperature was typically increased to 60 °C. All further NMR experiments were recorded on a Bruker AVANCE NEO 600 MHz spectrometer equipped with a 5 mm SmartProbe^{TM} set to a probe temperature of 65 °C.

Diffusion-Edited ^{1}H Experiments

The diffusion-edited ^{1}H experiment used a 1D bipolar-pulse pair with stimulated echo (BPPSTE)(12) diffusion-ordered spectroscopy (DOSY) pulse sequence (Bruker pulse program ledbpgp2s1d), with 1 s relaxation delay (d1), 0.5 s acquisition time (aq), 16 dummy scans (ds) and 128 transient scans (ns), a sweep-width (sw) of 20 ppm with the transmitter offset on 6.1 ppm (o1p), a diffusion time (d20) of 200 ms, a gradient recovery delay (d16) of 0.2 ms, an eddy current delay (d21) of 5 ms, a diffusion gradient pulse duration (p30) of 2.5 ms and a z-gradient strength (gpz6) of 90% at $\geq$ 50 G/cm (probe z-gradient strength). These conditions are specific to the Bruker AVANCE NEO 600 MHz - SmartProbe™ system and may need reoptimization for other systems. The diffusion-edited ^{1}H spectra for the 7, 20 and 30 wt% water samples are shown in **Supplementary Figure 17**, alongside a ^{1}H spectrum of cellulose triacetate (CTA) in $CDCl_3$ for comparison.

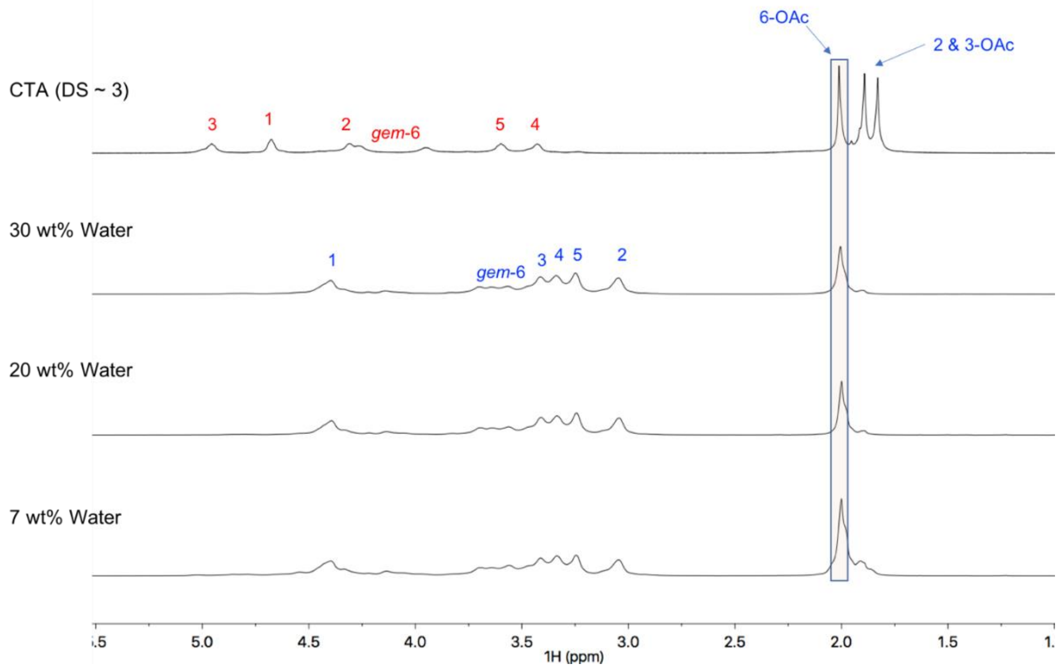

Supplementary Figure 17: Diffusion-edited spectra (in $[P_{4444}][OAc]$:DMSO-d_6 at 65 °C) for 7, 20 and 30 wt% water samples, in comparison to CTA (in $CDCl_3$ at 25 °C).

Multiplicity-Edited HSQC Experiment

The HSQC experiments used a multiplicity-edited phase sensitive HSQC sequence with echo/antiecho-TPPI gradient selection (Bruker pulse program hsqcedetgp)(13). The parameters are as follows: spectral width (sw) was 13.03 and 165 ppm, with transmitter offsets (o1p) of 6.18 and 75 ppm, for ^{1}H and ^{13}C dimensions, respectively. The time-domain size (td1) in the indirectly detected ^{13}C-dimension (f1) was 512, corresponding to 256 t1-increments for the real spectrum. There were 16 dummy scans (ds), 64 scans (ns), an acquisition time (aq) of 0.065 s for f2 and a relaxation delay of 1.5 s. Spectral resolution was increased by zero-filling, by setting the size of the spectrum in both dimensions to 1024 Hz. Sine squared (90 o) window functions were used in f1 and f2. The HSQC spectrum for the 7 wt% water sample is shown in **Supplementary Figure 18**.

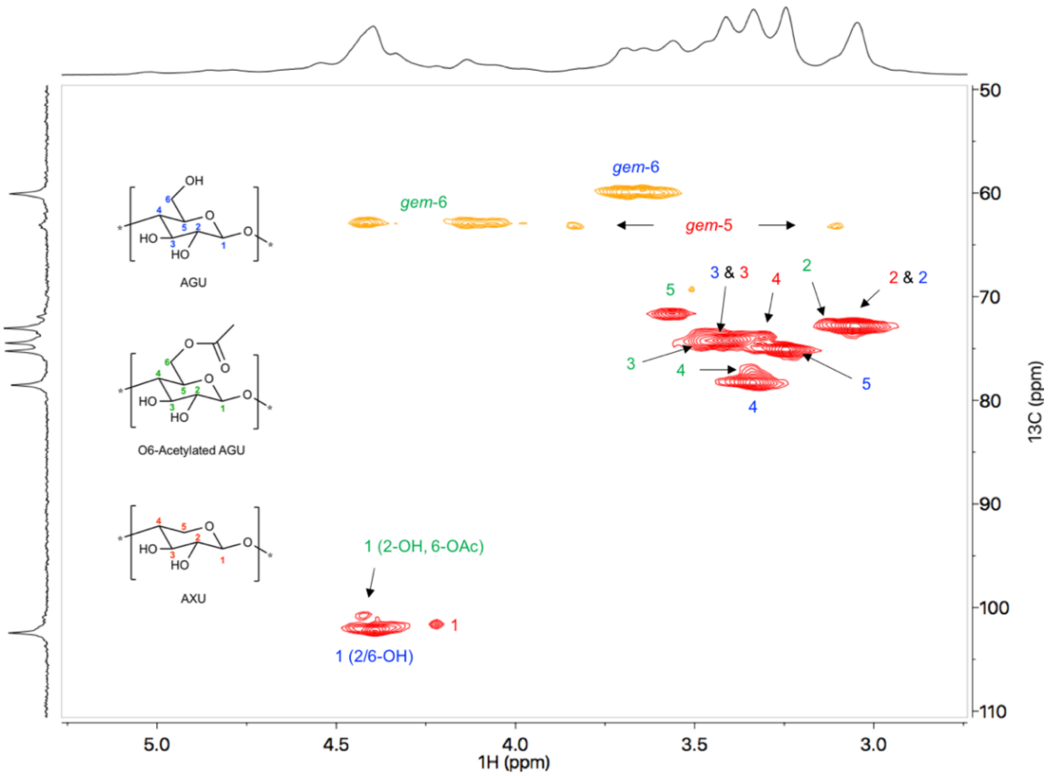

Supplementary Figure 18: Multiplicity-edited HSQC spectrum of the 7 wt% water sample in $[P_{4444}][OAc]$:DMSO-d_6 at 65 °C. Monoacetylation (to 6-OAc) is clearly evident.

HSQC-TOCSY Experiments

The HSQC-TOCSY experiment used a phase sensitive HSQC-TOCSY pulse program with the DIPSI-2 isotropic mixing sequence and echo/antiecho-TPPI gradient selection (Bruker pulse program hsqcdietgpsisp.2)(13). The parameters are as follows: spectral widths (sw) were 13.0 and 200 ppm, with transmitter offsets (o1p) of 6.18 and 90 ppm for ^{1}H and ^{13}C dimensions, respectively. The time-domain size (td1) was 512 in the indirectly detected ^{13}C-dimension (f1) dimension. There were 16 dummy scans (ds), 48 scans (ns), an acquisition time (aq) of 0.107 s for f2 and a relaxation delay of 1.5 s. The TOCSY mixing delay (d9) was 0.015 s to yield a short-range (COSY-like) TOCSY experiment. Spectral resolution was increased by zero-filling, by setting the size of the spectrum in both dimensions to 1024 Hz. Sine squared (90°) window functions were used in f1 and f2. The HSQC-TOCSY and overlaid HSQC spectra for the 7 wt% water sample (EMC) are shown in **Supplementary Figure 19** (full cellulose region) and **Supplementary Figure 20** (expanded 2-5 region), with the 6-OAc (monoacetate) spin-system correlations traced.

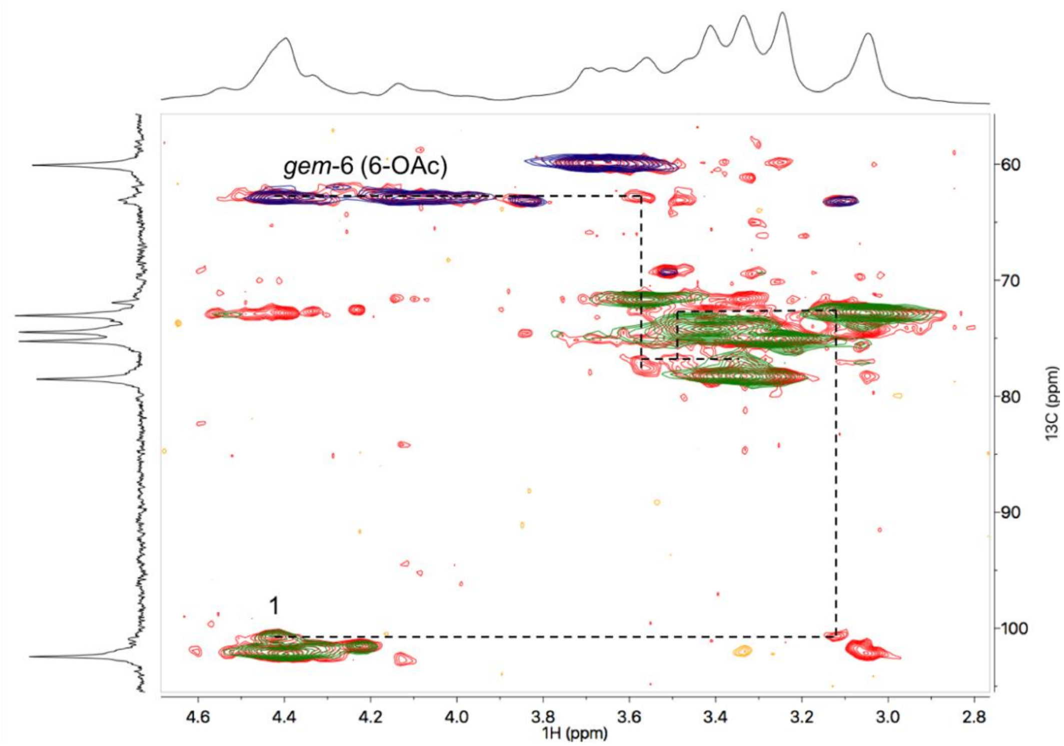

Supplementary Figure 19: HSQC-TOCSY (CH 1-6 region, full cellulose backbone) with overlaid HSQC for the 7 wt% water sample in [P_{4444}][OAc]:DMSO-d_6 at 65 °C.

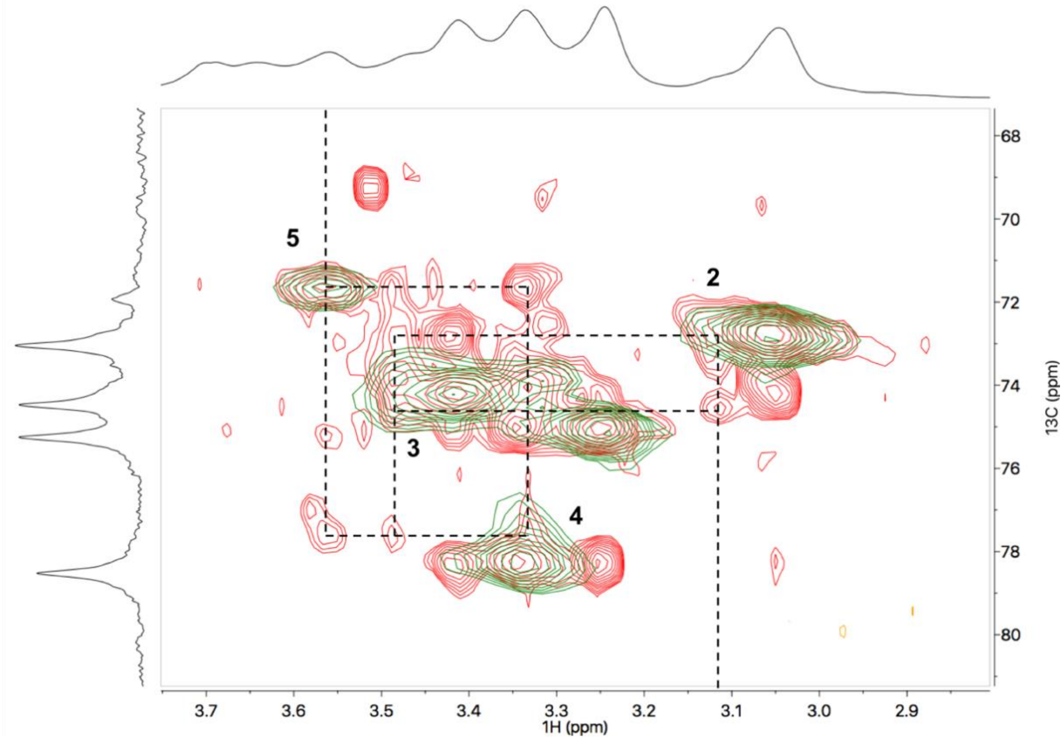

Supplementary Figure 20: HSQC-TOCSY (CH 2-5 region) with overlaid HSQC for the 7 wt% water sample in [P_{4444}][OAc]:DMSO-d_6 at 65 °C.

Regioselectivity Determination

The regioselectivity of acetylation was determined from quantitative ^{1}H spectra. The raw data was opened in MestreNova 10 (https://mestrelab.com/). The spectra were calibrated, phased and baseline corrected (3rd order polynomial). The xy data were output as NMR CSV files and opened in fityk(*14*) as .xy files. The full chemical shift region, from 1.5 – 5 ppm was fitted with pseudoVoigt functions (90% Lorentzian character). Care was taken to fit functions representing the water signal and ionic liquid signals, which were overlapping with the cellulose backbone signals and acetate signals, respectively. This was required as there was no further, more aggressive, baseline correction applied to get rid of these contributions. Manual manipulation of the peaks was performed to fit the spectra. Data fitting algorithms were not applied, after the initial fitting guesses. DS was then calculated from the peak volumes representing the acetate signals vs the cellulose backbone signals, weighted for the number of protons in those signals. The regioselectivity was calculated from the 6-Acetate peak volumes vs the total acetate peak volumes (**Supplementary Figure 21**).

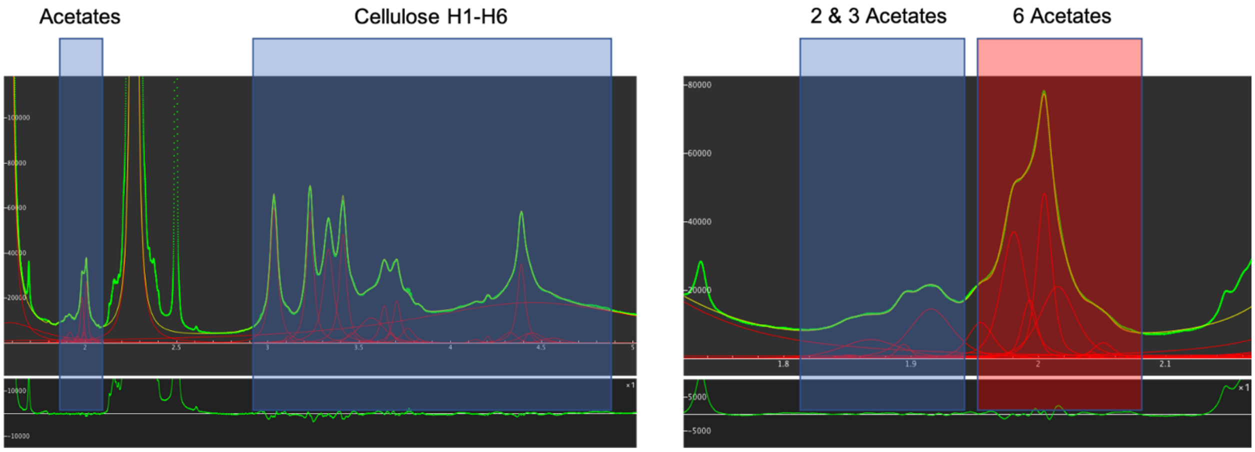

Supplementary Figure 21: Deconvolution of the quantitative ^{1}H spectra using pseudoVoigt functions for calculation of DS and regioselectivity.

Supplementary Section 1: Calculation of Transition States

The transition states (TS) for acetylation of Methanol (MeOH) using different combinations of MeOH, *N*-acetylimidazole (AcIm), imidazole (Im) and water (H_2O) (**Supplementary Figure 22**) were located through relaxed potential energy surface (rPES) scans and transition state searches (OptTS) in ORCA 4(15). The two combinations of interest, to test the effect of water in Im (base) catalysed esterification with AcIm were: **Combination A**) MeOH-AcIm-Im and **Combination B**) MeOH-AcIm-H_2O-Im.

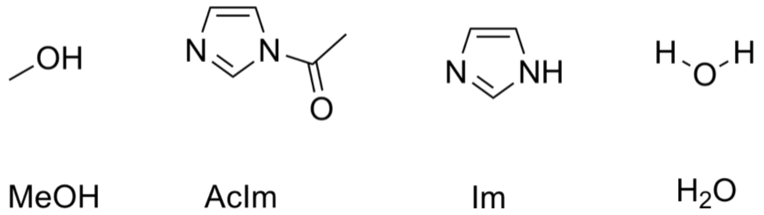

Supplementary Figure 22: Structures of the species that are used in the rPES and TS searches.

Initial structures (**Supplementary Figure 22**) were generated in Avogadro 1.2*(16)* and initially minimised using the mmff94 forcefield*(17)*. Rough low energy conformers for the combinations were located. rPES scans were then performed, by varying bond-lengths, typically between the MeOH oxygen and AcIm carbonyl carbon. The ORCA 4 package was used with the BP86 GGA functional(*18*, *19*), def2-SVP basis set, Grimme's D3 dispersion correction(*20*) with Becke-Johnson dampening(*21*) and the resolution-of-identity (RI) approximation(*22*, *23*). The start and end points for the reactions were determined by minimizing the low energy points in the rPESs. The transition-states were calculated (OptTS), using the approximate TS points from the rPES scans. Finally, the Gibbs free energies (NumFreq) were determined for all (start, TS & end point) geometries, after optimisation.

For both combinations, A) MeOH-AcIm-Im and B) MeOH-AcIm-H_2O-Im, transition states could be approximated and calculated, and the respective reaction coordinate energy diagrams are shown in **Supplementary Figures 23-24**.

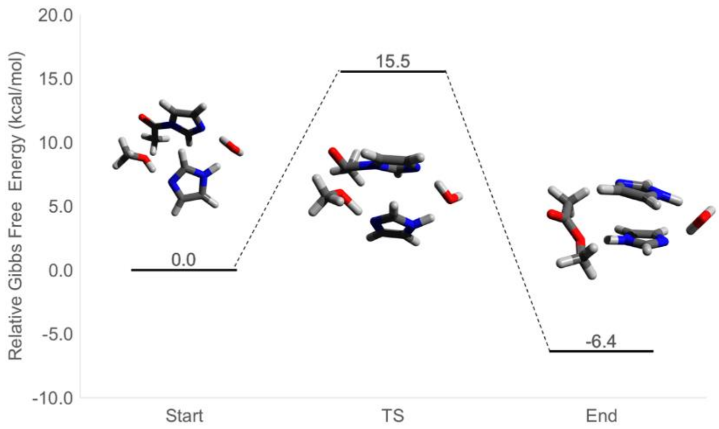

Supplementary Figure 23: Reaction coordinate energy diagram for the **MeOH – AcIm – H_2O – Im** (Combination B).

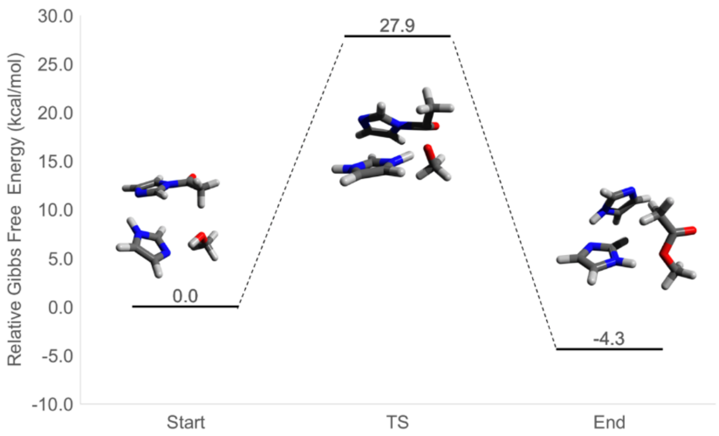

Supplementary Figure 24: Reaction coordinate energy diagram for the **MeOH – AcIm – Im** (Combination A).

Clearly having a small amount of water in the reaction is advantageous for esterification kinetics (due to reduced strain during proton transfer). This seems to be a singular feature of imidazole, as a leaving group, in acyl activation. Other typical leaving groups, e.g. chloride (acyl chlorides) and acetate (mixed anhydrides), allow for direct proton transfer to the leaving species, in the absence of additional waters (**Supplementary Figure 25**).

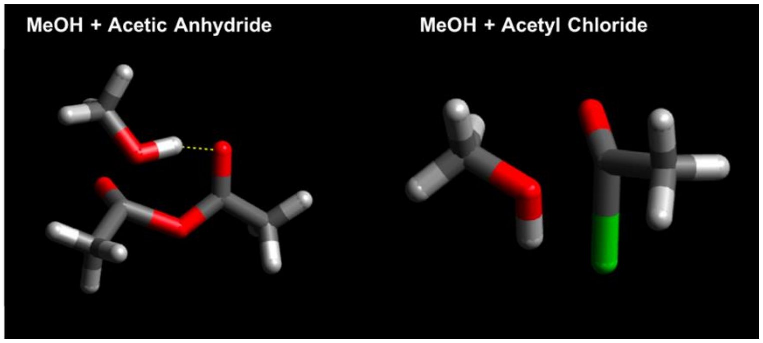

Supplementary Figure 25: Comparison of the transition states for acetylation of methanol using acetylating agents containing different leaving groups (acetate and chloride). Concerted proton transfer at the transition state is possible for all these combinations, without additional water or imidazole.

The Combination B transition state was also modelled on a cellulose Iβ surface fragment. To achieve this, an initial cellulose Iβ fibril (hexagonal 36 chain) with a polymer length of 4 glucose units was generated using the Cellulose-Builder(*24*) web interface (http://cces-sw.iqm.unicamp.br/cces/admin/cellulose/view;jsessionid=). This was then edited in Avogadro to remove all polymer chains except for a (110) surface section of 3 stacked polymer chains with a length of 4 AGUs. Hydroxyl groups were added to the reducing ends, as these are missing in the Cellulose-Builder outputs. An acetate was added to a 6-OH of a central AGU. A rPES for a dihedral angle scan of the corresponding C-C-C-O(-Ac) angle was completed at the HF-3c level(*25*) throughout the full 360° (**Supplementary Figure 26**); constraints were used on all atoms except the 6-OAc, all oxygens, all 1,2,3,4-hydrogens attached to OHs and the 6-CH_2 positions attached to 6-OH and 6-OAc.

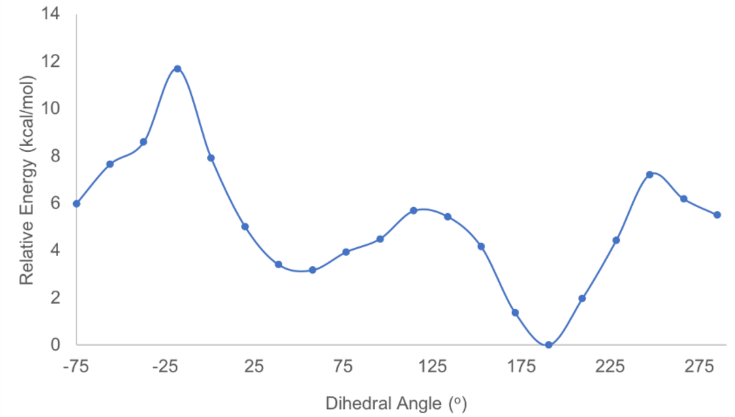

Supplementary Figure 26: rPES dihedral scan for C-C-C-O(Ac) at the 6 position on a cellulose Iβ surface fragment.

This prevented movement of the AGUs away from the geometry found in the typical cellulose Iβ crystalline structure but allowed for enough freedom for formation and breakage of H-bonds, necessary for stabilising conformers. The conformer at 58° was then further minimised at the HF-3c level and subsequently at RI-BP86/def2-SVP-D3(BJ) level, to get comparable energies with the MeOH model. Thermochemical calculations were performed to get the Gibbs free energy values (**Supplementary Figure 27** and **Supplementary Table 3**). The optimised geometries used for the calculation of the transition states can be found in the appended **Data S1**.

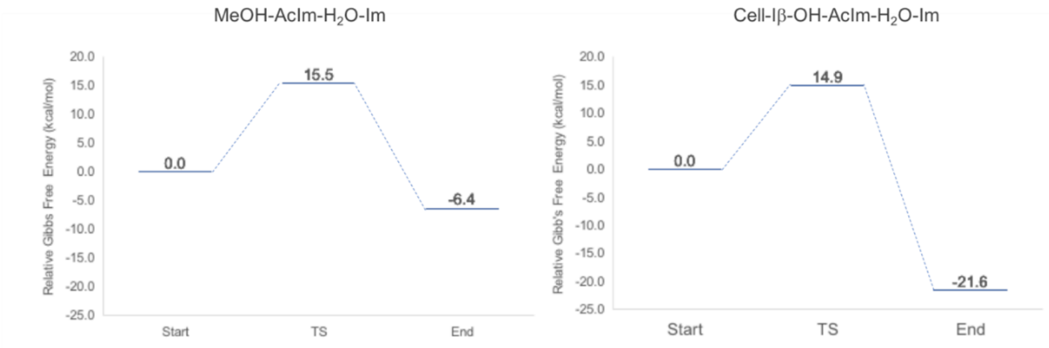

Supplementary Figure 27: TS enthalpies for the **MeOH – AcIm – H_2O – Im** and **Cell-Iβ-OH – AcIm – H_2O – Im** combinations at the RI-BP86/def2-SVP-D3(BJ) level.

Supplementary Table 3. Gibbs free energies for intermediates, transition states and products, relative to the starting material combinations.

Combination	Computational Level	ΔG^{TS} (kcal/mol)[a]	ΔH^{TS} (kcal/mol)
MeOH - AcIm - Im	RI-BP86/def2-SVP-D3(BJ)	27.9[b]	23.0[b]
MeOH - AcIm - H_2O - Im	RI-BP86/def2-SVP-D3(BJ)	15.5	11.7
Cell-Iβ-OH - AcIm - H_2O - Im	RI-BP86/def2-SVP-D3(BJ)	14.9[c]	12.4[c]

[a] at room temperature. [b] this is the barrier height from the rPES (bond length) scan, as a OptTS calculation could not locate the TS. [c] this is the barrier height from from the rPES (bond length) scan, as a OptTS calculation was too computationally expensive.

Supplementary References

1. H. Sixta, Ed., *Handbook of pulp* (Wiley-VCH, Weinheim, Germany, 2006).

2. Y. Okita, T. Saito, A. Isogai, Entire Surface Oxidation of Various Cellulose Microfibrils by TEMPO-Mediated Oxidation. *Biomacromolecules*. **11**, 1696–1700 (2010).

3. K. Wickholm, P. T. Larsson, T. Iversen, Assignment of non-crystalline forms in cellulose I by CP/MAS 13C NMR spectroscopy. Carbohydrate Research. 312, 123–129 (1998).

4. A. Paajanen, S. Ceccherini, T. Maloney, J. A. Ketoja, Chirality and bound water in the hierarchical cellulose structure. *Cellulose*. **26**, 5877–5892 (2019).

5. D. P. Oehme, M. T. Downton, M. S. Doblin, J. Wagner, M. J. Gidley, A. Bacic, Unique Aspects of the Structure and Dynamics of Elementary Iβ Cellulose Microfibrils Revealed by Computational Simulations. Plant Physiology. 168, 3–17 (2015).

6. X. Kang, P. Sun, S. Kuga, C. Wang, Y. Zhao, M. Wu, Y. Huang, Thin Cellulose Nanofiber from Corncob Cellulose and Its Performance in Transparent Nanopaper. ACS Sustainable Chem. Eng. 5, 2529–2534 (2017).

7. T. Wang, M. Hong, Solid-state NMR investigations of cellulose structure and interactions with matrix polysaccharides in plant primary cell walls. J Exp Bot. 67, 503–514 (2016).

8. D. Massiot, F. Fayon, M. Capron, I. King, S. Le Calvé, B. Alonso, J.-O. Durand, B. Bujoli, Z. Gan, G. Hoatson, Modelling one- and two-dimensional solid-state NMR spectra. Magn. Reson. Chem. 40, 70–76 (2002).

9. R. H. Newman, Estimation of the lateral dimensions of cellulose crystallites using 13C NMR signal strengths. Solid State Nuclear Magnetic Resonance. 15, 21–29 (1999).

10. A. W. T. King, V. Mäkelä, S. A. Kedzior, T. Laaksonen, G. J. Partl, S. Heikkinen, H. Koskela, H. A. Heikkinen, A. J. Holding, E. D. Cranston, I. Kilpeläinen, Liquid-State NMR Analysis of Nanocelluloses. *Biomacromolecules*. **19**, 2708–2720 (2018).

11. Koso, T. et al. 2D Assignment and quantitative analysis of cellulose and oxidized celluloses using solution-state NMR spectroscopy. Cellulose 27, 7929–7953 (2020).

12. D. H. Wu, A. D. Chen, C. S. Johnson, An Improved Diffusion-Ordered Spectroscopy Experiment Incorporating Bipolar-Gradient Pulses. Journal of Magnetic Resonance, Series A. 115, 260–264 (1995).

13. W. Willker, D. Leibfritz, R. Kerssebaum, W. Bermel, Gradient selection in inverse heteronuclear correlation spectroscopy. Magnetic Resonance in Chemistry. 31, 287–292 (1993).

14. Wojdyr, M. *Fityk* : a general-purpose peak fitting program. *J Appl Crystallogr* **43**, 1126–1128 (2010).

15. F. Neese, Software update: the ORCA program system, version 4.0. WIREs Comput Mol Sci. 8 (2018), doi:10.1002/wcms.1327.

16. M. D. Hanwell, D. E. Curtis, D. C. Lonie, T. Vandermeersch, E. Zurek, G. R. Hutchison, Avogadro: an advanced semantic chemical editor, visualization, and analysis platform. J Cheminform. 4, 17 (2012).

17. T. A. Halgren, Merck molecular force field. I. Basis, form, scope, parameterization, and performance of MMFF94. Journal of Computational Chemistry. 17, 490–519 (1996).

18. A. D. Becke, Density-functional exchange-energy approximation with correct asymptotic behavior. Phys. Rev. A. 38, 3098–3100 (1988).

19. J. P. Perdew, Density-functional approximation for the correlation energy of the inhomogeneous electron gas. Phys. Rev. B. 33, 8822–8824 (1986).

20. S. Grimme, J. Antony, S. Ehrlich, H. Krieg, A consistent and accurate ab initio parametrization of density functional dispersion correction (DFT-D) for the 94 elements H-Pu. The Journal of Chemical Physics. 132, 154104 (2010).

21. S. Grimme, S. Ehrlich, L. Goerigk, Effect of the damping function in dispersion corrected density functional theory. J. Comput. Chem. 32, 1456–1465 (2011).

22. K. Eichkorn, O. Treutler, H. Öhm, M. Häser, R. Ahlrichs, Auxiliary basis sets to approximate Coulomb potentials (Chem. Phys. Letters 240 (1995) 283-290). Chemical Physics Letters. 242, 652–660 (1995).

23. K. Eichkorn, F. Weigend, O. Treutler, R. Ahlrichs, Auxiliary basis sets for main row atoms and transition metals and their use to approximate Coulomb potentials. Theoretical Chemistry Accounts: Theory, Computation, and Modeling (Theoretica Chimica Acta). 97, 119–124 (1997).

24. T. C. F. Gomes, M. S. Skaf, Cellulose-Builder: A toolkit for building crystalline structures of cellulose. J. Comput. Chem. 33, 1338–1346 (2012).

25. R. Sure, S. Grimme, Corrected small basis set Hartree-Fock method for large systems. J. Comput. Chem. 34, 1672–1685 (2013).

Data S1: Optimised geometries (DFT level)

The optimised geometries for the low molecular weight fragments (RI-BP86/def2-SVP-D3(BJ) level) are as follows:

Combination A: MeOH – AcIm – Im (Start)

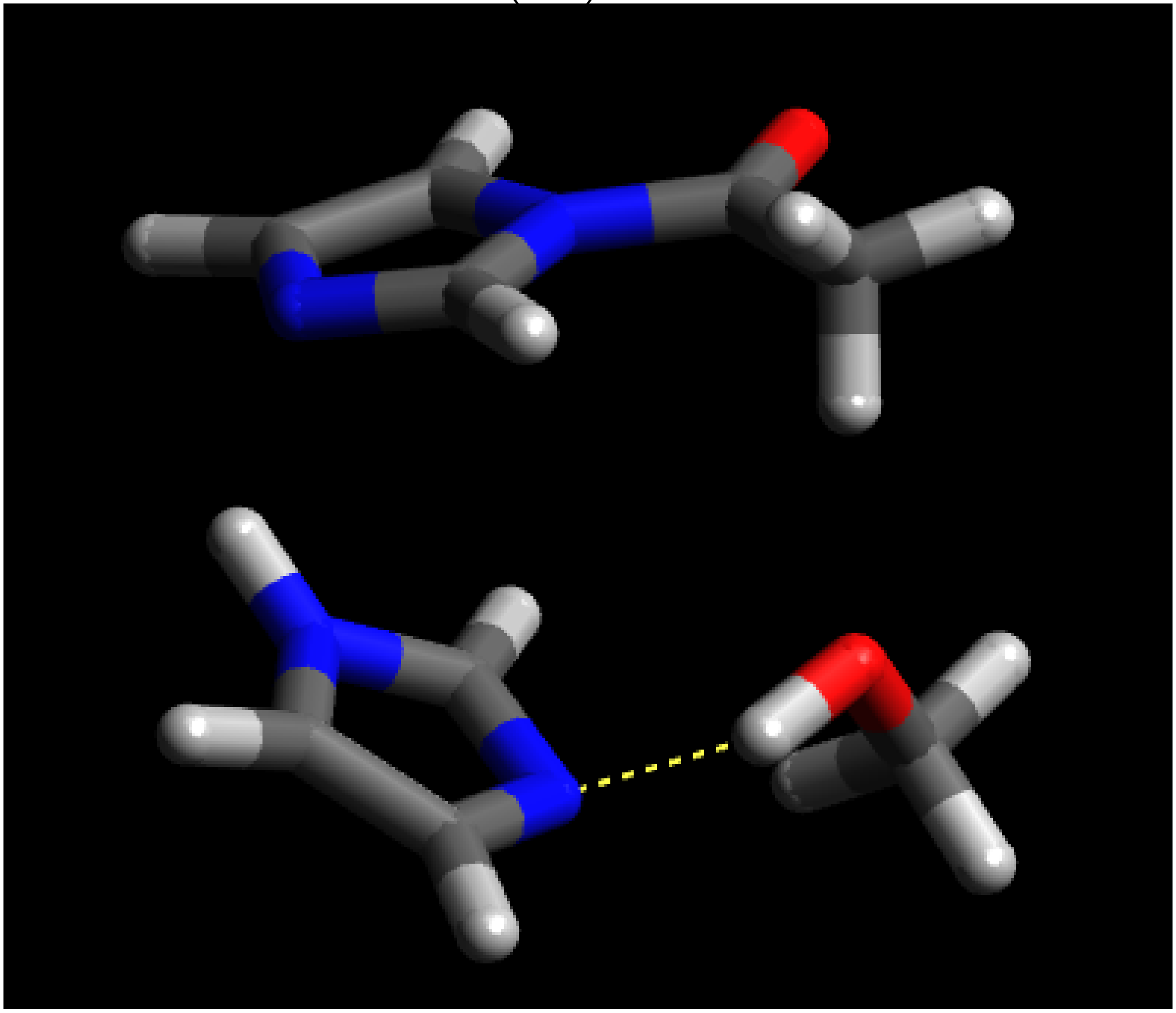

Supplementary Figure 28: Combination A: MeOH – AcIm – Im (Start)

29

Coordinates from ORCA-job orca

C	-1.74438970087590	-0.77294578830365	0.92958454564812
O	-0.84854923874715	-0.73046440608062	-0.15943961796490
H	-0.47513247047990	-1.65053425886746	-0.29078739199179
C	1.59994563287554	2.29819614085196	0.52572347320654
O	1.32416669333245	3.19552889386685	1.29657498944224
C	0.83217914134369	1.93509574245269	-0.71281196541556
H	0.12773049090785	2.75554901740914	-0.93573498642402
H	0.24942644392298	0.99341635862027	-0.53812060938784

H	1.49720294184128	1.76862384494441	-1.58305538501034
N	2.73028229762336	1.44846877111990	0.83014705467101
C	3.46906349201352	1.51852338717892	2.01200862280522
C	4.36040430982673	0.46763430380107	1.95247338641125
N	4.19265306768727	-0.24523576898150	0.76973394106026
C	3.21991145951971	0.36818036258414	0.11662338407658
H	2.80605907817753	0.06380970996066	-0.85056670812769
H	3.26091920602343	2.29768364128348	2.75074221944717
H	5.11358053251898	0.17604586642749	2.69455019080491
N	0.74034187216157	-2.95526661507844	-0.18365844916698
C	1.42133573960356	-3.88263243346498	-0.94890892854930
C	1.67672088984402	-2.28475618880743	0.48300653284504
N	2.92832937458640	-2.73619656691565	0.18372294235631
C	2.78880769850029	-3.75592092862756	-0.73970671165434
H	3.78068883928053	-2.20849254427437	0.45487767329158
H	0.89633953801193	-4.58411549553560	-1.60848784969119
H	1.48994020431957	-1.44626067854210	1.16260565413697
H	3.64911859157601	-4.28741881827674	-1.15816263968635
H	-2.06380957137604	0.26418186422950	1.16321674311435
H	-1.29243971727334	-1.19944133654181	1.85842736884143
H	-2.66728683674583	-1.36252607643256	0.71049252091124

Combination A: MeOH – AcIm – Im (TS)

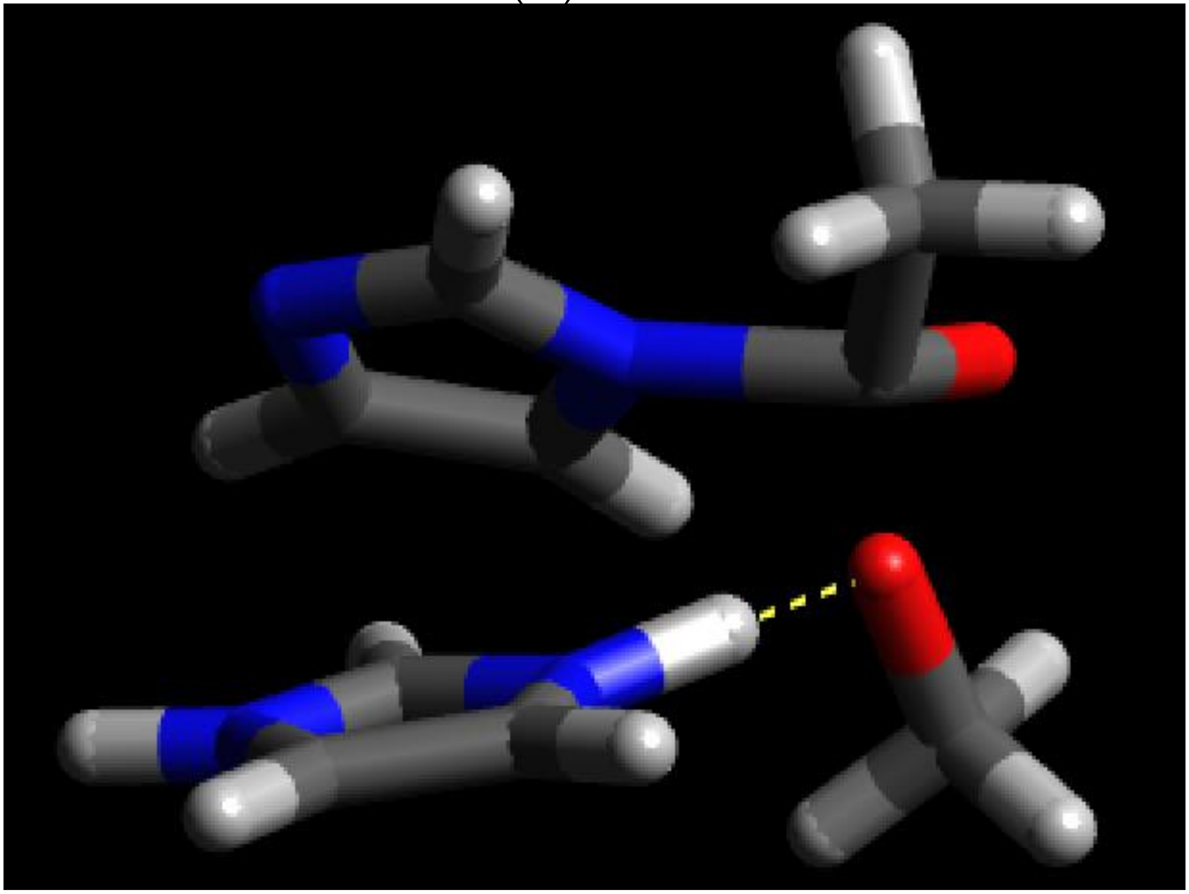

Supplementary Figure 29: Combination A: MeOH – AcIm – Im (TS)

29

Coordinates from ORCA-job orca

C	-0.93358292685692	-0.26037212690096	1.39813151913527
O	-0.13246071985799	-0.01351276087457	0.23779610748214
H	0.59869041188880	-1.08988517868877	0.00236850603820
C	0.54266858679650	1.31606973932451	0.19932229804908
O	0.05779614707946	2.16106337705947	0.97398037048098
C	0.68753630683255	1.61856340050323	-1.29969081837785
H	-0.31777395827367	1.86768311435546	-1.69027982872214
H	1.09412239587939	0.76812637701876	-1.88503455081787
H	1.34664005093826	2.49757097593220	-1.42620810749636
N	2.09614991998497	0.87385987994578	0.69316604459112
C	2.42086293570176	0.84714182640823	2.02812841618070
C	3.75661298054075	0.43854836152295	2.09708684828408
N	4.25783421816437	0.22843036075763	0.82789623857304
C	3.22767258144258	0.50358733503102	0.01483180823895
H	3.26533616093582	0.44689321478261	-1.08135404956576
H	1.69125434119469	1.17326882678244	2.77775374312611
H	4.38359670394574	0.31688463951281	2.99156753478727

N	1.28935316289013	-2.05398473835509	-0.07454200808359
C	1.30345754063782	-3.06852712996255	-1.01275009618130
C	2.37298983998518	-2.17039549866655	0.69880413064338
N	3.07276189048975	-3.25402672622958	0.29107285248328
C	2.42897926457611	-3.83620320840250	-0.79228983394069
H	3.97519647071743	-3.53150506850299	0.68125704445801
H	0.51858793957191	-3.17214563624283	-1.76867914574582
H	2.65109291847617	-1.50092306457156	1.53133995929590
H	2.81435817694044	-4.72654968029816	-1.29615243916831
H	-1.12828896787219	0.72863889593519	1.86106171855753
H	-0.39865115433922	-0.91528908446060	2.12218348598353
H	-1.87925321841055	-0.74428042271568	1.08430225171105

Combination A: MeOH – AcIm – Im (End)

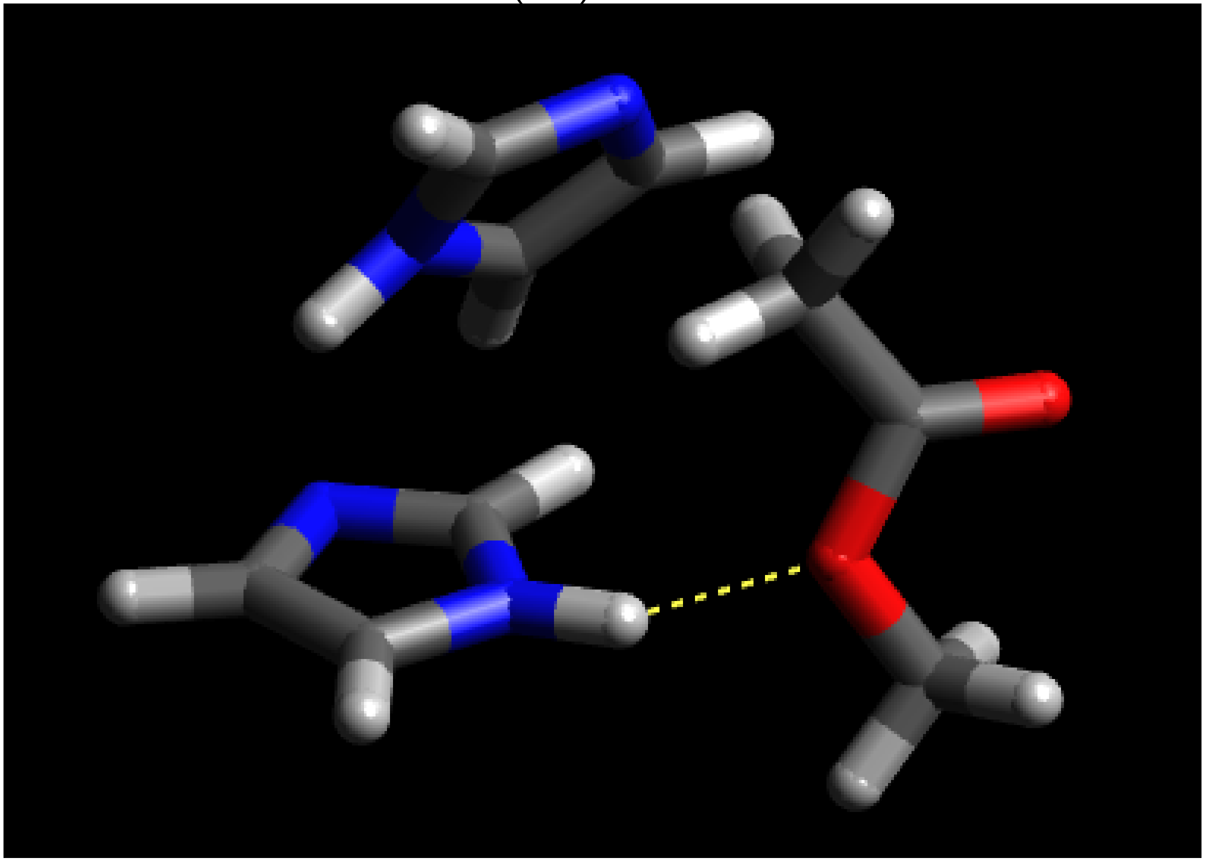

Supplementary Figure 30: Combination A: MeOH – AcIm – Im (End)

29
Coordinates from ORCA-job orca

C	-1.74009007400327	-0.30259113540015	1.48178487734095
O	-0.77355623329305	-0.09623446424401	0.43497388101188
H	-0.05011011590267	-1.76417268082326	-0.37260097037004
C	-0.46439284189172	1.23138132792766	0.18219023167854
O	-1.01017179683765	2.13092677343891	0.78355078342003
C	0.60836215319479	1.37235769599585	-0.86186982613187
H	0.35946299288428	2.22820752820552	-1.51689877424647
H	0.74479154260335	0.45335638889399	-1.46034316273005
H	1.57561663772491	1.61511271238764	-0.36239780164400
N	3.75577515233053	1.58400054897954	0.51222289092312
C	3.81839293570059	1.36122090755735	1.87511663504611
C	4.22680871602448	0.05766359292255	2.13299099135684
N	4.44247383694253	-0.51400458100252	0.89168952837469
C	4.12569711568768	0.43426442109121	-0.04432680840060
H	4.16332920424836	0.22341954703093	-1.12109868792095
H	3.57241116988911	2.14898283323285	2.59859073020003
H	4.38912373962735	-0.49659614178390	3.06313843062090
N	0.81986981613842	-2.31332352124621	-0.38611353341106

C	1.16062173050557	-3.42052873941867	-1.14134122663397
C	1.91949799436641	-1.94812147157945	0.33369519444292
N	2.95038110080088	-2.75539913141549	0.09045419393935
C	2.48824289002245	-3.68063411448900	-0.82585335219927
H	4.42780068787366	-1.53365041630880	0.68385229408789
H	0.45265462753493	-3.91139064695122	-1.81665941551778
H	1.91805462223191	-1.08188020124439	1.00717863527956
H	3.13064696607789	-4.48072442993127	-1.21341447247140
H	-2.71352505761579	0.15355908806761	1.21306601107572
H	-1.39110659897298	0.15043533287446	2.43044008625097
H	-1.84352291389333	-1.39690702276775	1.59305263662803

Combination B: MeOH – AcIm – H_2O – Im (Start)

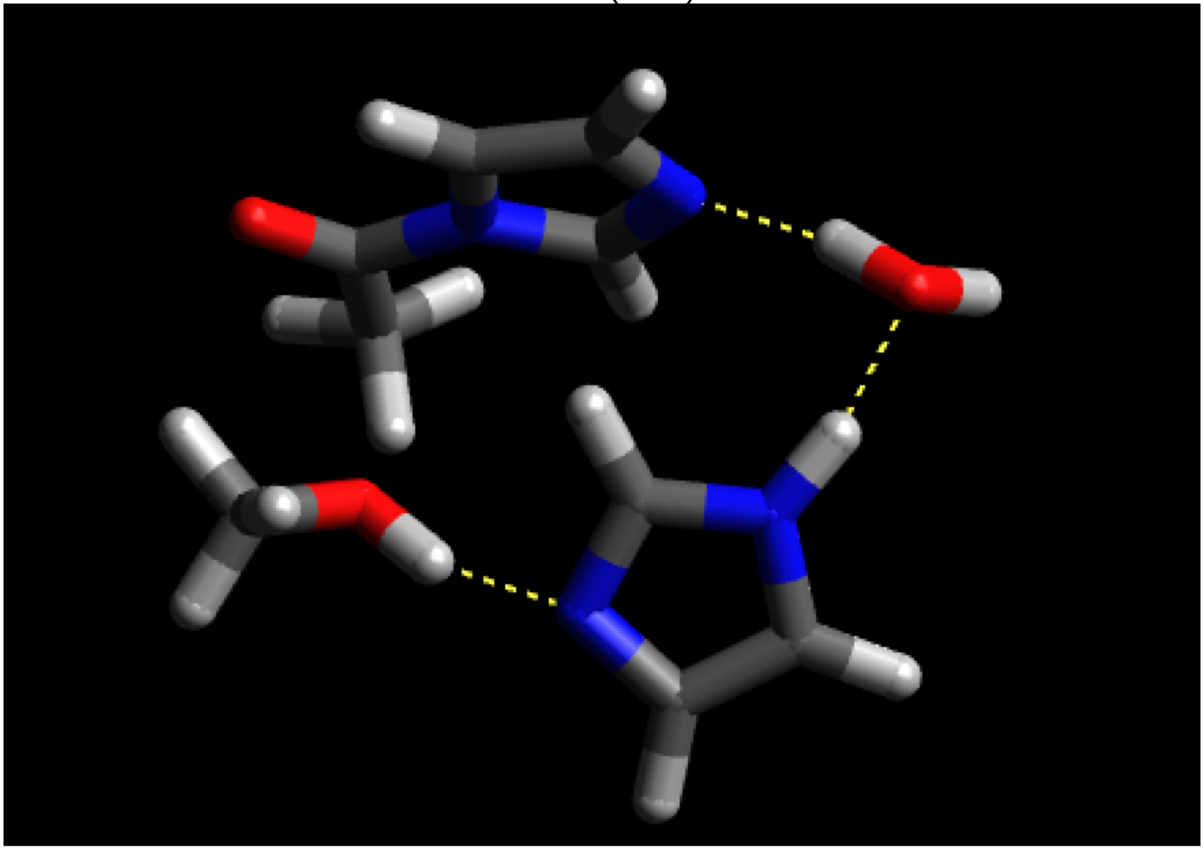

Supplementary Figure 31: Combination B: MeOH – AcIm – H_2O – Im (Start)

32
Coordinates from ORCA-job orca

C	-1.32900225928799	-0.38619757913529	1.36428801947624
O	-0.40555098365135	-0.48517781453328	0.30598376478390
H	-0.19906535735322	-1.45886122013884	0.14015452846296
C	1.14972319137171	1.81377963041709	0.30167445605695
O	0.53649598093141	2.39597620767468	1.17140784195090
C	0.80987238931802	1.82091745774122	-1.16326293200519
H	0.11944281185211	2.66166853164611	-1.35308020122019
H	0.27143767290731	0.86910584121164	-1.35608628099651
H	1.69768122963908	1.90325235111110	-1.81859882418908
N	2.33032482216409	1.06097296729350	0.67862254637883
C	2.76523833857375	0.93928487075721	1.99480181790651
C	3.89206558661337	0.14656803185639	1.94381420684363
N	4.15519338709110	-0.22065342359199	0.62927790670883
C	3.20165301376036	0.33212871589074	-0.10583925034547
H	3.07565659346981	0.21491419628029	-1.18775090613391
H	2.22093430887065	1.43512734831833	2.80292495413975
H	4.53407679846319	-0.18620866514955	2.76765337290367
O	5.44292871506698	-2.60649929553493	0.34194503673758
H	5.90526691183668	-2.64081237481856	-0.51675382971994

H	5.10064250195988	-1.65438228640681	0.38870206964009
N	0.68831307165900	-2.90660700850976	-0.03090278874444
C	0.90271454881100	-3.94506169915820	-0.91354333862329
C	1.88282704759227	-2.62037442721825	0.48894182697093
N	2.85621224557789	-3.42920042937781	-0.01382064827047
C	2.25089250719020	-4.28099320982288	-0.91777348006756
H	3.89305559891700	-3.28939036230290	0.15439616729373
H	0.08534456730350	-4.39197530454759	-1.49196231613557
H	2.07693445449714	-1.83456240367616	1.22783502404752
H	2.81466468355459	-5.04009384893595	-1.47014364061136
H	-1.34212594876665	0.66620348967706	1.71957628879252
H	-1.06181496257930	-1.03255288877755	2.23476938599092
H	-2.37347346735369	-0.65220539823905	1.06399922197752

Combination B: MeOH – AcIm – H_2O – Im (TS)

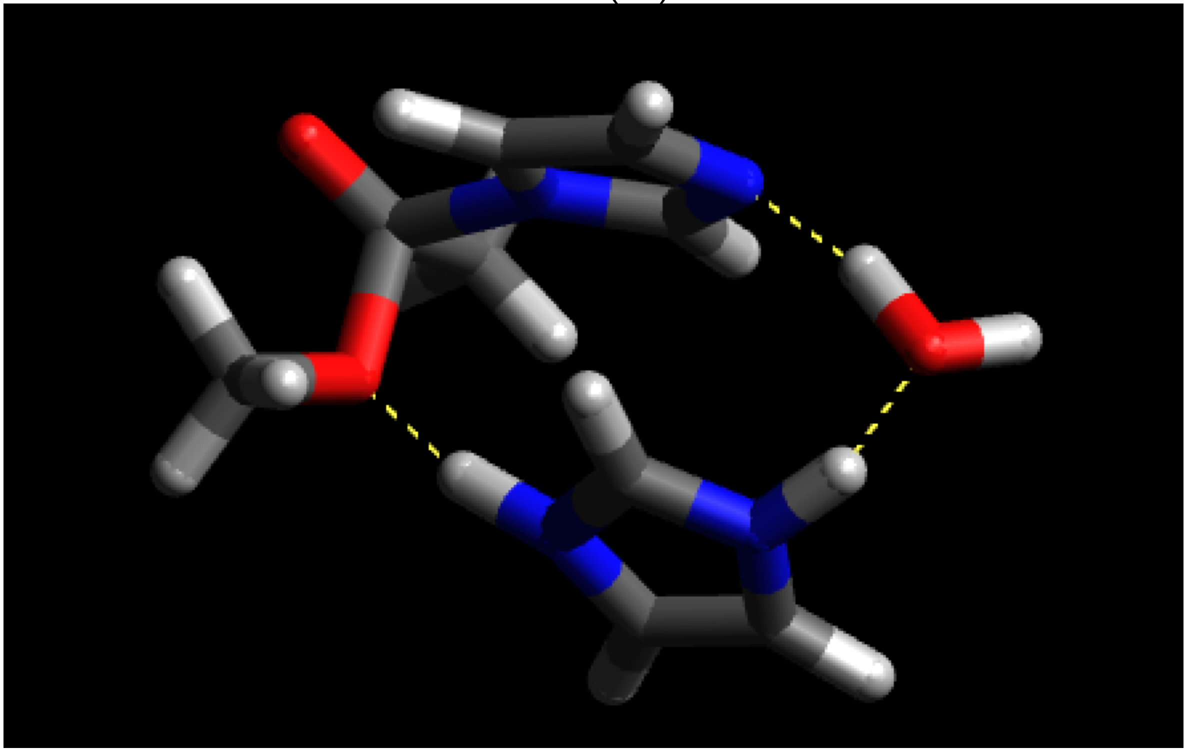

Supplementary Figure 32: Combination B: MeOH – AcIm – H_2O – Im (TS)

32
Coordinates from ORCA-job orca

C	-0.93085774132251	-0.09861513697019	1.69951709103522
O	-0.32282047158551	-0.09461085000851	0.41511424661938
H	0.22228097670032	-1.39560924610757	0.08287215939717
C	0.49570124558742	1.22331806032157	0.09978226388222
O	0.20359517009958	2.18836333059476	0.78714009842174
C	0.61202642434726	1.22904523038890	-1.41251639275814
H	-0.37480915235981	1.49965617478475	-1.83665860263790
H	0.91322425214912	0.24338290603404	-1.81616238856073
H	1.35281009533028	1.99294990483231	-1.71353593919668
N	2.16957602637797	0.61064031045419	0.60754863435216
C	2.50582613730116	0.64255811558896	1.93797408446675
C	3.74387148829418	0.01068184478169	2.06493841233519
N	4.17346793693131	-0.39439482143507	0.81602543852208
C	3.19094694503195	-0.01222754557197	-0.03045247179774
H	3.20424008954026	-0.19615203445069	-1.11299468554993
H	1.85571804180988	1.13940427788598	2.66723825764633
H	4.34733136964568	-0.16412008948582	2.96464042933198
O	5.26707619755970	-2.76532203238251	0.56219228538867
H	5.88066958379718	-2.78578973202508	-0.19638221223661
H	4.98707431186397	-1.75455987655059	0.64793590478020
N	0.85874436725481	-2.30028956986429	-0.09799777601564

C	0.96761040519865	-3.07927257753268	-1.23659105159874
C	1.98336027993416	-2.45179213246799	0.61876314081247
N	2.80185236527318	-3.32330809878435	0.00175803235469
C	2.19036328066564	-3.72211200157833	-1.17552445953879
H	3.88192489324608	-3.31283658145291	0.23692756503794
H	0.17579960656990	-3.12117013832419	-1.99120127259379
H	2.21707247100033	-1.91053302195111	1.54633104688552
H	2.66095295530421	-4.42758184746681	-1.86731360183378
H	-1.03308494627887	0.95292596935277	2.04018079792771
H	-0.31947209945654	-0.65706826334455	2.44621748544902
H	-1.93351250581167	-0.56747052726439	1.62548347967196

Combination B: MeOH – AcIm – H_2O – Im (End)

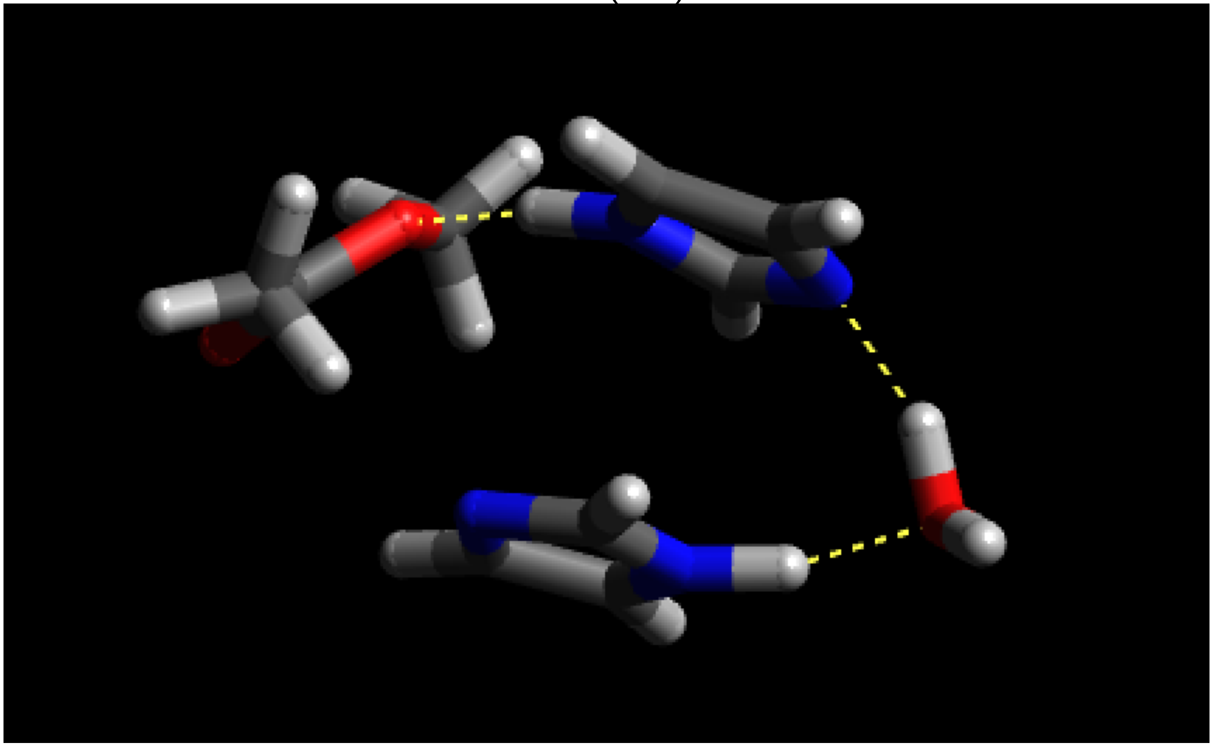

Supplementary Figure 33: Combination B: MeOH – AcIm – H_2O – Im (End)

32

Coordinates from ORCA-job orca

C	-0.80794457222437	-0.11758964198451	2.01255142881933
O	-0.54570000910111	0.07475765527818	0.61086571935047
H	0.37525883080521	-1.31264107205564	-0.12106453658168
C	-0.38716318232677	1.39344215286123	0.21036378175063
O	-0.53438342975358	2.31166152368960	0.98826957022828
C	0.03070190521441	1.47232113253517	-1.22788466641093
H	-0.46179051704416	0.69516150315576	-1.84250201165479
H	1.13747320645567	1.29826165887604	-1.23636510997641
H	-0.18768736975372	2.48045406998715	-1.62232326887666
N	2.94666418024912	0.71418802961569	-0.20005313810865
C	2.85760417078129	0.93500911742628	1.16119544976464
C	3.63179952272519	0.00258376669519	1.84509888919231
N	4.20859807909602	-0.79015967558761	0.87327871823585
C	3.75690528194307	-0.33414588784364	-0.33315918039645
H	4.02977287673369	-0.81447262445532	-1.28110581590233
H	2.26098392585561	1.75948096028850	1.57210979369961
H	3.83243209383630	-0.14985432417726	2.91108101246914
O	5.08546292835694	-3.36584220988369	1.16346488675600
H	5.75236179631070	-3.67121884237259	0.52122379070801
H	4.71150124373858	-1.70916851268395	1.02528972983099
N	1.01695871707239	-2.10219547572046	-0.32669602340027

C	1.33844294040642	-2.61867218243987	-1.56578773550514
C	1.82062173879032	-2.70499538227897	0.59266264272116
N	2.63591742227005	-3.59023588465837	0.01796112230367
C	2.34454476297694	-3.54552743769511	-1.33237366169326
H	4.20573855280374	-3.67272393442132	0.76411247706415
H	0.83760762772428	-2.29100894789594	-2.48149306539020
H	1.80658864008340	-2.44033723924962	1.65645664611064
H	2.86762978211808	-4.17886981584068	-2.05888340180781
H	-1.64649267759573	0.52453111613066	2.34322947735431
H	0.09294105997948	0.13162838984164	2.60956516357944
H	-1.06478952852835	-1.18573198513616	2.13216131576584

The optimised geometries for the cellulose Iβ surface fragment (RI-BP86/def2-SVP-D3(BJ) level) are as follows:

Cell-Iβ-OH – AcIm – H_2O – Im (Start)

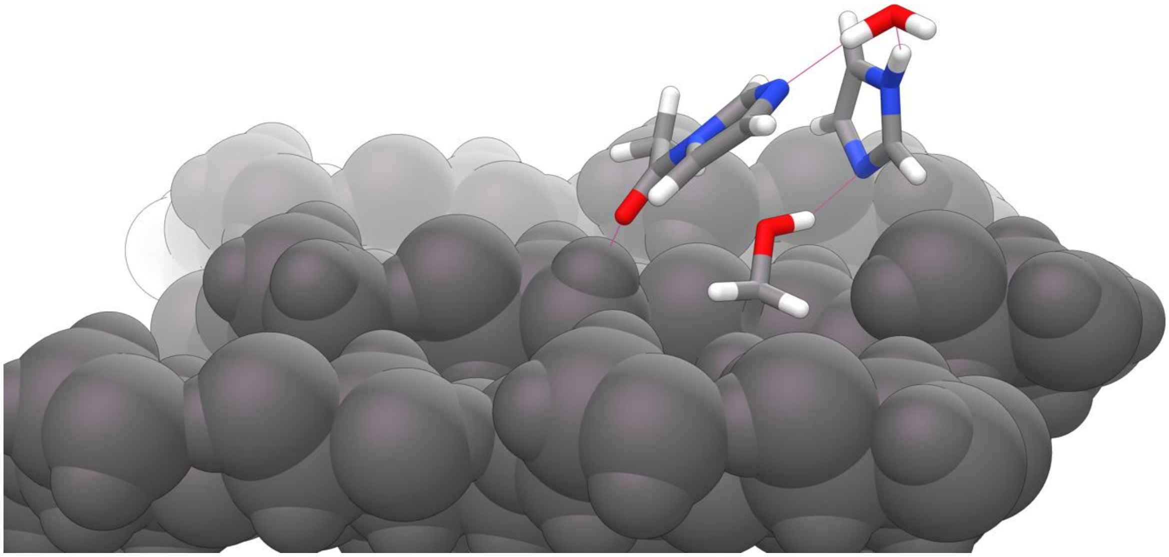

Supplementary Figure 34: Cell-Iβ-OH – AcIm – H_2O – Im (Start)

287
Coordinates from ORCA-job orca

C	19.23343016147476	3.71552077470098	3.16691886835561
H	20.30609980674728	3.77883917692866	3.36623121474135
C	18.92928007250099	2.58102995449252	2.18043996220774
H	17.85228999738722	2.39039995311092	2.16356005138126
C	19.35188994344392	2.94280003340224	0.76294000881109
H	20.40923999591039	2.96465000160846	0.63965999807668
C	18.81575001562229	4.30980996679288	0.36277996221620
H	17.73681999887331	4.20884999716237	0.21843001019087
C	19.12620990618255	5.36443017053281	1.44018009446389
H	20.19894995265799	5.58348982978223	1.44841999499872
C	18.34932999838436	6.65545993522277	1.15673003401723
H	17.33060999853197	6.41083000086094	0.83641000294504
H	18.26340000065270	7.24253000305824	2.07684000415650
O	19.64225849980121	1.41330373237178	2.55125777845542
O	18.82404978856941	1.96457317870382	-0.13461892266920
O	19.44539580106012	4.72084202498142	-0.85049303857830
O	18.68614460658674	4.95106665215735	2.73309827883135
O	18.92586961500280	7.38191884378163	0.06935310530832
H	19.38893365125366	1.17846999634038	3.48126893911453
H	19.10530713174868	1.10694460451083	0.24223011674253
H	19.76176757957232	7.79576295985137	0.37455898512209
C	22.51997992691534	7.86374032986306	0.44024934768077

H	23.58635996400619	8.05468983198661	0.61908043208950
C	22.39085021198282	6.71213005870095	-0.56470006277830
H	21.37433999304712	6.31164993681245	-0.57650978521674
C	22.87110984747304	7.07474008177001	-1.94658009261411
H	23.96941001558962	7.07301996586947	-1.95997997646640
C	22.31139005889251	8.41664998324573	-2.37778992491758
H	21.24828999261466	8.28925002642594	-2.61260004425048
C	22.47590004636220	9.46190936037905	-1.26087986993629
H	23.54196990490987	9.66230070327114	-1.09963006976666
C	21.77893001066763	10.76919003778991	-1.61250002635974
H	20.74205000290315	10.57048998890460	-1.90877999103585
H	21.74673000145554	11.40637999983784	-0.72398000187256
O	23.19895776267542	5.59615507973317	-0.13984277049283
O	22.42316007929469	6.12659572842746	-2.92039397903903
O	22.99243636061469	8.91754953739323	-3.53245532414561
O	21.84878054921976	9.00561364234780	-0.04814414768508
O	22.49883530622408	11.47742729492538	-2.60733697531227
H	22.76306451444843	5.22570433965588	0.65205412474486
H	22.68516211595943	5.24474108893885	-2.59179607748659
H	22.68999954879086	10.81956938004130	-3.31249308958662
C	26.11995008409799	11.86512996139362	3.16892992850747
H	27.20165998844539	11.93418999635763	3.34484001276673
C	25.82213994499933	10.73484020361476	2.18163031513202
H	24.74502998018436	10.55881979487948	2.13195985533636
C	26.32663995651037	11.10873978433272	0.79156983685137
H	27.42220004095267	11.16070001566456	0.78085001846113
C	25.71790004616624	12.45503000887215	0.37545001747623
H	24.63556998490077	12.32598998461452	0.23878998606527
C	26.00105997750762	13.51812999837638	1.44992000861898
H	27.07495999452050	13.74860999985062	1.45842000281306
C	25.20906999469370	14.79783999883883	1.15020000469548
H	24.18815999853744	14.54963000039175	0.83747000184932
H	25.11627000294642	15.39762999962709	2.06125999863151
O	26.47817798835810	9.55726344667346	2.62704981576958
O	25.90442765631118	10.12535175413075	-0.16511949348128
O	26.28657446950206	12.94879802002720	-0.83884575547887
O	25.56317877584395	13.09239984530315	2.73864341767332
O	25.86458485218234	15.57989188456450	0.16809227692803
H	26.11140649527876	9.32504313523548	3.52099749921989
H	26.14334272242711	9.25264702675929	0.21254433243695
C	22.30687965394575	8.49991042990034	5.65849085634541
H	21.24552007129802	8.28271965368023	5.82868927520970
C	22.43702008335543	9.66220008046889	4.66995058637080
H	23.47526019806999	9.99796984191248	4.62245928565320
C	21.95800996358487	9.27432010063260	3.27107994541072
H	20.86353000594161	9.24016996753764	3.25126000993344

C	22.54910997550025	7.92624998192075	2.84619005185921
H	23.62281001185350	8.03992004064700	2.65822998068101
C	22.35337003701738	6.91791996775414	3.99581998327472
H	21.28750992847092	6.69819025444251	4.13094995321947
C	22.99840554808247	5.56938492713219	3.63409422741949
H	22.78684389922146	4.85811189575394	4.46081092722822
H	22.48205150460288	5.17699180843392	2.72268060588703
O	21.62773752155218	10.73622771584632	5.13165409615993
O	22.39000186830831	10.30972579243722	2.38169256896217
O	21.92157957406296	7.44547956086678	1.64058149868671
O	22.99514349355409	7.37760211388413	5.15956863412875
O	24.39280963452167	5.62897880769931	3.48882051380416
H	21.98471984262297	10.97769110150756	6.02004978742829
H	22.03942413039882	10.08560621106426	1.48908465971021
C	25.34770763324851	4.47480490450783	5.79527231153028
C	18.72707998645319	4.43236997858413	8.35972998598921
H	17.64540999333893	4.36083003482646	8.53470000798118
C	19.02502999192953	5.56449967868538	7.37387993543014
H	20.10251008388804	5.73761050101174	7.32359022406558
C	18.52409002642246	5.19348987143576	5.98447993448344
H	17.42865999274882	5.13587003551403	5.97015999545363
C	19.14031998367048	3.84880020175058	5.56907041858587
H	20.22678016665775	3.97462968381974	5.45949956491294
C	18.84934008878158	2.78410007161888	6.64008995241139
H	17.77498994270323	2.55238998452427	6.64634001276976
C	19.58372939610463	1.45117903167661	6.36394024244877
H	20.65357818693466	1.65520987699983	6.15732458506527
H	19.53549321654110	0.83653373097730	7.29251799021317
O	18.35669821956459	6.73401899214630	7.81368106969162
O	18.95381919764567	6.23034740192552	5.10352972719699
O	18.57921124165036	3.37061762656205	4.35340826894113
O	19.29136398088545	3.19597115282595	7.91537817257995
O	19.08827802947148	0.72336048864229	5.24458808496111
H	18.66986554024261	6.93649284439815	8.73225823398686
H	18.70216250122533	5.95446686095904	4.18937932145162
H	18.13512441188026	0.56273097269615	5.38084588325762
C	25.58818000672545	12.58768995862369	8.36217000638070
H	24.50991000561373	12.52163005591655	8.54425999865663
C	25.89372999809845	13.70199000478512	7.36127001123391
H	26.97790000120190	13.86237000055119	7.31790999844043
C	25.37533000518320	13.32637998948515	5.98204998288517
H	24.27990999902170	13.26883001775810	5.97365000788803
C	26.00358996092197	11.99295005274407	5.56659010802716
H	27.08824998851636	12.12484999974578	5.46345997604927
C	25.71244005988691	10.93284000275698	6.64148975704281
H	24.64188997492643	10.70943991155281	6.64814018485007

C	26.39646313701997	9.57221116455433	6.36396159242617
H	27.46236667457305	9.72635236262187	6.09710161106281
H	26.37150111997039	8.99004706142675	7.31362608080202
O	25.23886766468507	14.87317623094558	7.84246306851126
O	25.77830234562897	14.39918613552527	5.13577686158468
O	25.46773385341504	11.50659397071091	4.35099350678936
O	26.15750657884531	11.35906127247228	7.91702992612470
O	25.81478016357655	8.82940490766174	5.29768660752326
H	25.56376464873250	14.13984186110205	4.20432542057697
H	24.92175919980929	8.50506292776306	5.55429281941365
C	19.25739000063424	3.71558997381866	13.54451000476395
H	20.33536999577954	3.78280003150066	13.73081000020036
C	18.96503999776349	2.58336999940618	12.56039999640732
H	17.88281999886885	2.40407000210745	12.51415000047100
C	19.47021999983641	2.95812999541034	11.17255999771384
H	20.56565000248936	3.01761000006608	11.16018000253682
C	18.84514999427874	4.29869002320474	10.75849998245358
H	17.76004999827826	4.16953999752003	10.65473999921966
C	19.13532000725731	5.36255995997275	11.82842005902173
H	20.20579997301958	5.58671007356931	11.83614994080185
C	18.34993000146205	6.64921003812875	11.53608996051279
H	17.32957000123210	6.39988000059620	11.22617000282112
H	18.26985000127308	7.24687000022232	12.45040000155066
O	19.66844829789588	1.43704646207296	12.99710687718625
O	19.04685186463710	1.92098293226261	10.29799523760293
O	19.38452895081367	4.77582145032177	9.53796158666803
O	18.69454373456409	4.94731250756142	13.11025388252870
O	18.91487206074468	7.43981589093180	10.49067760148894
H	19.37033429434464	1.22511271692599	13.91699284147450
H	19.29722907596205	2.19899341866124	9.38658625882169
H	19.78783002154293	7.77496746628601	10.78517075142934
C	22.53143998120058	7.83455975069821	10.82294925952762
H	23.59490999542529	8.03634016204927	11.00214982970755
C	22.40688007519368	6.65347005741450	9.85342992918867
H	21.37218000593602	6.30592990366184	9.80962006711404
C	22.88809981616425	7.03835989503893	8.46012982250683
H	23.98287004191459	7.06352999541197	8.44137002520392
C	22.29863016246672	8.38963010615634	8.02989011278766
H	21.22561999368421	8.26468985431855	7.84413988025026
C	22.47558982999223	9.44503040042361	9.12939040819052
H	23.54195015347495	9.65438955742637	9.28017953416455
C	21.77141998838084	10.75757996017384	8.77202998464886
H	20.73225000722579	10.55921000221308	8.48933998064798
H	21.75545001557679	11.40851999226400	9.65153000225442
O	23.23257049287158	5.60650514744088	10.34062160289821
O	22.42137040312441	6.05738208837142	7.54272809104175

O	22.92363499030689	8.88209651984286	6.85279407214895
O	21.86586118017888	9.00671389061520	10.34227000491558
O	22.46674334935683	11.45817791384613	7.72468963371919
H	22.86110859609040	5.33504367435625	11.21526543425914
H	23.16856817577649	5.48034178182312	7.26488412726483
H	22.16059149553227	12.38499528131884	7.74500325553591
C	26.12065999188855	11.86153001369225	13.54481999369389
H	27.20221000079496	11.93311000231417	13.72135999832719
C	25.82281006431824	10.73087990250696	12.55969001992857
H	24.74519997819405	10.55775008638739	12.51119996753356
C	26.32398989880571	11.10342013580284	11.17153015821956
H	27.41913000752749	11.16325998644394	11.15936996528447
C	25.70315001727899	12.44786000409871	10.75982000290018
H	24.61726999722043	12.32050999550305	10.65200999647529
C	25.99711999942152	13.51075999462040	11.83290000446077
H	27.07205999955396	13.74061999995097	11.83836999905547
C	25.21174000020826	14.79465000177235	11.53134999794913
H	24.19115999912926	14.55407999927732	11.21002999834186
H	25.11485999893610	15.39065999975673	12.44426000045192
O	26.47263038067106	9.55134104855591	13.00528304808993
O	25.89261281115367	10.06128765665455	10.28852293766065
O	26.24388865856625	12.93956474755147	9.53871303910517
O	25.55455946012811	13.08891126832497	13.11427050020812
O	25.85705313466348	15.63095696369951	10.59334031499652
H	26.12510272161088	9.34435205057711	13.91058691011130
H	26.13760428702959	10.34436647193186	9.37571071167169
C	22.22383998686844	8.45909999549192	15.97586001257098
C	22.40805998210697	9.64894002395775	15.03087003528954
H	23.45669000679896	9.95827998602469	14.99912995015197
C	21.94670001334607	9.26594997957499	13.63161000289948
H	20.85294000361262	9.23965000502922	13.60640999766567
C	22.53997996105955	7.91098002820803	13.20422997919966
H	23.61551999855934	8.03285998125720	13.02622999912106
C	22.36087008164994	6.85008985232004	14.30277012943275
H	21.29650994283738	6.62870016320867	14.44269986199474
C	23.09652356151403	5.53649885556425	14.00902510110122
H	24.15035970343683	5.75696805367219	13.72268282183242
H	23.11140843116157	4.96046094300047	14.95976916829455
O	21.64850406466107	10.72238920366716	15.58757245107562
O	22.37328914133932	10.31484365350265	12.75553728025323
O	21.90811999951013	7.45510540527666	12.01009772763590
O	22.92810036474838	7.33419697847058	15.51008181520142
O	22.42423986531586	4.80168199642490	12.97631479967313
H	21.82722867756425	11.49939560954539	15.02353664983850
H	22.06519332962738	10.07616289351308	11.84825645551676
H	22.74584186921009	3.88045057481880	13.00294756522283

C	18.65218000175612	4.43251999713371	18.68929999738270
C	19.00101998733648	5.56902000412095	17.72952999248266
H	20.08905000050644	5.71781999881353	17.70012999698015
C	18.51443003441682	5.19684994547480	16.33900005345753
H	17.41944000016236	5.13962000223130	16.32171998205672
C	19.13618999249841	3.84687999311622	15.93549000279106
H	20.22368000041083	3.97440999884489	15.83557999777537
C	18.84339999909677	2.78068999979469	17.00614000319318
H	17.77027000222910	2.54287999874605	17.00843000033199
C	19.58454606580493	1.45131468752517	16.75467486451116
H	20.65107986207937	1.65199648066649	16.52664391302980
H	19.55105941279384	0.87180489791721	17.70594370115510
O	18.35420788195490	6.75490781705080	18.15650096387028
O	18.95222796360625	6.22976503114897	15.45681022143429
O	18.59135205044345	3.36708238798853	14.72097226157713
O	19.25989604549412	3.21537567058816	18.28782827873497
O	19.07842184955256	0.69479404041871	15.66216053564206
H	18.53146713475452	6.80773418517967	19.11821656321371
H	18.72210357400570	5.94466006935230	14.53753534278422
H	18.12864443829774	0.52919604597013	15.81426968682140
C	25.50858999933595	12.58264999767745	18.70099999579904
C	25.86081999956326	13.71005000010568	17.72955999897180
H	26.94981999984526	13.84960999979903	17.70298999822703
C	25.36857000248835	13.33357000402454	16.34138000304388
H	24.27345999620627	13.27451000059795	16.32527999918955
C	26.00181000878552	11.99579999146673	15.93517000166836
H	27.08732999659452	12.12649000649365	15.83735999485255
C	25.70804999366512	10.93638000748328	17.00641003303880
H	24.63536000608534	10.71759000240458	17.00907996966725
C	26.38808382010793	9.57780170673387	16.73018930646249
H	27.44107431846799	9.73527285497333	16.41676336638674
H	26.40395514395320	9.02562583677773	17.69648146067145
O	25.23603011734031	14.91034572674171	18.15330754476856
O	25.77833929066359	14.40637912683375	15.49469556512063
O	25.46551295672956	11.50411071087978	14.71890996894314
O	26.13552287073183	11.37438808201452	18.27611820002727
O	25.76578193668869	8.81581406639858	15.70233742118994
H	25.57060217138335	14.14526099886402	14.56353601947904
H	24.90171530888711	8.48267021271696	16.02983789483671
H	24.42292000028418	12.47615000232607	18.83228999873721
O	25.95742042585172	12.84981720642568	19.99577914986427
H	17.56731000003960	4.32562000059237	18.82682999978823
O	19.11461809794686	4.73637440245048	19.98346997386141
H	20.09212052463414	4.77041544603112	19.92895945857521
H	26.88920025732551	13.13640672566438	19.92851483578394
H	21.16256000255148	8.23140000274902	16.13267999781336

O	22.82807019810055	8.75724439192446	17.22126756372442
H	22.53569313004628	9.67088840041060	17.42583527758850
H	19.21462555843278	5.67106816658530	-0.97042473884048
H	22.89225597799435	8.23621866037909	-4.22621825695055
H	26.01896480627080	12.32644816621087	-1.54299857330303
H	26.02153579184236	14.96471110087526	-0.58131030397196
H	25.72503029748789	15.25963913472350	9.69106762483293
H	25.28022291875316	15.49110303223568	17.36513953603187
H	25.32289348519556	15.53435421739157	7.12431638032744
C	26.31523639646596	5.61559841334262	5.89128169203175
O	24.34011808440636	4.35557635644797	6.47969620775552
H	26.01717800805319	6.38016326960000	5.14536323723617
H	27.36049879854551	5.31442889975650	5.69564792883622
H	26.23266797041082	6.06020462221381	6.89911739840218
H	27.42459365543418	7.65687361784808	2.40742187076671
H	29.16563937700851	5.55180642964002	1.70922670791725
C	27.22277683165093	6.71933023364700	1.87930269220358
C	28.09472828193521	5.69604185346124	1.53140971240791
N	25.95624791817082	6.43854244160032	1.38727155080743
H	24.70377353883541	6.08985199791304	2.65398596629906
N	27.34446995546745	4.78638036544034	0.80833345476498
C	26.07289373507616	5.26414840916170	0.75464336938963
H	27.62683043010117	3.79257953318211	0.56463182898423
H	25.24893913793385	4.74807476360102	0.25059743787802
O	27.82919918721051	2.13377349152505	0.70541584659204
H	27.25485241936003	1.63543978278133	0.09454527903359
H	27.32821839943257	2.10801090628495	1.58606448829219
H	27.20369878536686	4.32218580133112	3.60018212970790
N	25.63563553643743	3.44590804679460	4.84884963041819
C	26.53485236092018	3.47430914337365	3.79681492643218
C	24.88401232577965	2.27696006701695	4.71726035249114
H	24.09376350335513	2.03810872299052	5.43335173915457
N	26.40765057118579	2.39793871231242	3.04183576623184
C	25.38639167920329	1.63872170157977	3.60672569693455
H	25.08151517193183	0.67681179266302	3.17856108956829

Cell-Iβ-OH – AcIm – H_2O – Im (TS)

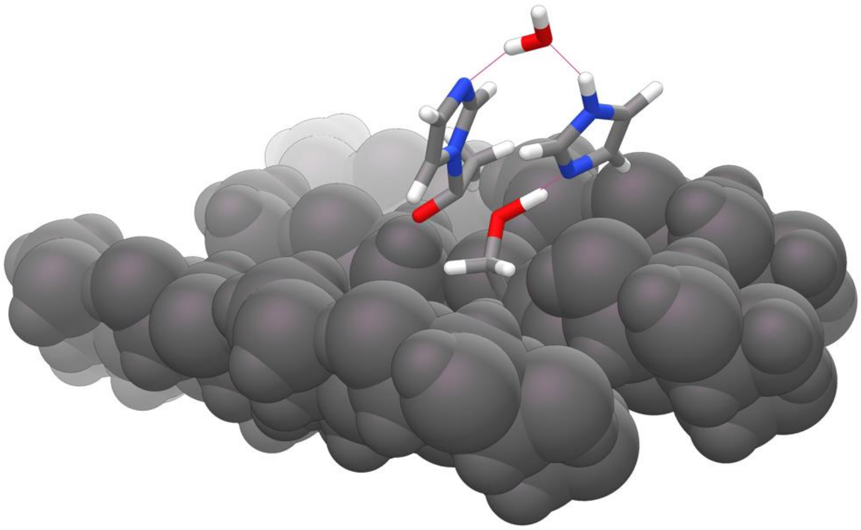

Supplementary Figure 35: Cell-Iβ-OH – AcIm – H_2O – Im (TS)

287
Coordinates from ORCA-job orca

C	19.23343030910064	3.71551963024037	3.16691846103371
H	20.30609988199752	3.77884072329634	3.36623215930300
C	18.92928004016928	2.58103009752023	2.18043986329132
H	17.85228996108632	2.39039996639350	2.16356000620350
C	19.35188995302297	2.94280001805139	0.76293998011099
H	20.40923999652289	2.96464997334161	0.63966002271322
C	18.81574991444826	4.30981028235895	0.36277978432208
H	17.73681999989103	4.20884996215629	0.21843000302843
C	19.12620995986229	5.36442970257037	1.44018033616051
H	20.19895025214079	5.58348993408909	1.44842023217646
C	18.34932997409427	6.65546004733037	1.15672999737407
H	17.33060994942487	6.41083002250522	0.83641000171513
H	18.26340000095478	7.24252999747213	2.07683999346076
O	19.64598481687058	1.41600011251339	2.54885554714163
O	18.82636488373416	1.96350792575785	-0.13443795833118
O	19.45165399623792	4.72193361550874	-0.84898908999920
O	18.68613192949079	4.95259752285749	2.73125172260078
O	18.92520395254846	7.38479181214693	0.07019031519959
H	19.39952464078007	1.17944066236902	3.48124837982283
H	19.09684209683509	1.10592350044698	0.25135814737753
H	19.75542055271050	7.80427536378623	0.38164542494325
C	22.51998002175363	7.86374046464825	0.44024925544477
H	23.58635995098295	8.05468976133542	0.61908049009847
C	22.39084982698513	6.71213043377769	-0.56469989911490
H	21.37434015049780	6.31164934092383	-0.57650996199382

C	22.87111001419962	7.07474003110834	-1.94658011207567
H	23.96940999860673	7.07301997361328	-1.95997997863547
C	22.31139003070363	8.41664995063992	-2.37778993246139
H	21.24828996957237	8.28925005275085	-2.61260002981805
C	22.47590011606476	9.46190973229752	-1.26087994955610
H	23.54196984683738	9.66230036050964	-1.09963003502137
C	21.77893003572964	10.76918995499508	-1.61249998655565
H	20.74204998960895	10.57049000056879	-1.90877999658762
H	21.74672999458409	11.40638000917146	-0.72397998826267
O	23.22148873320099	5.60658679064734	-0.14063011196070
O	22.42709608379351	6.12356778116934	-2.91724416177281
O	22.99266070819327	8.91946471627013	-3.53200695948475
O	21.85308560072428	9.00644209799043	-0.04718753423717
O	22.50018073746342	11.47780904445117	-2.60594079202345
H	22.68391657086416	5.06697584527823	0.46947883443250
H	22.70355344096657	5.24522406809987	-2.58982072527990
H	22.68581831287108	10.82265992543480	-3.31479240938710
C	26.11995014650078	11.86513013106885	3.16892982013942
H	27.20165996531682	11.93418993053704	3.34484005664121
C	25.82213979680132	10.73484029461565	2.18163017978753
H	24.74503003410063	10.55881957127581	2.13196007362043
C	26.32664000681285	11.10874032567443	0.79156977203609
H	27.42220005317051	11.16069981613676	0.78085004143625
C	25.71790003219573	12.45502983473431	0.37545004795025
H	24.63556997411068	12.32598998176321	0.23878999967387
C	26.00105998876970	13.51812999601368	1.44992000018025
H	27.07496000138298	13.74860998249202	1.45842000823638
C	25.20907000028202	14.79784000392658	1.15020000120819
H	24.18815999851750	14.54963000001184	0.83747000272199
H	25.11627000167154	15.39762999923781	2.06125999992538
O	26.48032027671289	9.54379970294741	2.61619650150024
O	25.89649439933162	10.14725372730135	-0.18741762724050
O	26.28749757309985	12.94526976706885	-0.83866762716315
O	25.56065657419485	13.08881194842321	2.73545498008054
O	25.86449850721311	15.58119789675204	0.16944066672978
H	26.16368288410300	9.33214776066345	3.53855767648956
H	26.24136969887837	9.27693748286405	0.09173659555763
C	22.30687937477901	8.49991038794409	5.65849093801491
H	21.24552018289401	8.28271965963598	5.82868927012302
C	22.43702012084879	9.66220039179901	4.66995048883232
H	23.47526032061316	9.99796959491747	4.62245955780842
C	21.95800988415144	9.27432012855426	3.27107997353132
H	20.86352999806062	9.24016989283187	3.25126002843868
C	22.54911014451434	7.92624988741537	2.84619006870188
H	23.62281001722760	8.03992003447212	2.65823003565254
C	22.35336966645269	6.91792008260880	3.99581886589833

H	21.28751010435168	6.69819021410745	4.13095066150720
C	22.99141022475891	5.55861753348379	3.69653014512434
H	22.62763184017506	4.82291467939932	4.44417385957629
H	22.71622224042459	5.22536760690466	2.67237867028125
O	21.63035615519029	10.73775270190256	5.12979560791866
O	22.39164151843322	10.30923138367854	2.37934431554048
O	21.92347566653225	7.44896099031538	1.64007489905803
O	22.98925869642655	7.38036230866160	5.16040008324221
O	24.39834327230579	5.64608858874570	3.81377301906401
H	21.98233131067766	10.97330075701560	6.02242415848173
H	22.03656920677735	10.08669412477951	1.48835977964742
C	24.98400683202751	4.87765179444674	4.97738306528607
C	18.72708003422776	4.43236984674419	8.35972985120037
H	17.64541000004258	4.36082997126287	8.53469998154219
C	19.02502962334832	5.56450157310866	7.37388086492801
H	20.10251041155125	5.73760835086727	7.32358916584097
C	18.52409008543829	5.19348991719705	5.98447980800860
H	17.42865996738125	5.13587000464189	5.97015994778672
C	19.14031990985639	3.84880111393033	5.56907109442328
H	20.22678032830595	3.97462825047908	5.45949835869666
C	18.84933974036918	2.78409992737764	6.64009030713944
H	17.77499012908850	2.55239008557703	6.64633992476336
C	19.59109160900373	1.45456476922192	6.36392118527073
H	20.66238741987403	1.66224042952549	6.16883295683724
H	19.53682192317801	0.83400925705807	7.28822217229852
O	18.35614560043017	6.73460873855028	7.81337063597380
O	18.95494049620154	6.22955370890597	5.10019005406051
O	18.57815811850032	3.37278802360701	4.35166221432142
O	19.29037956047464	3.19658514741967	7.91544272965526
O	19.10583397708299	0.73052533985266	5.23616660466740
H	18.67343286713517	6.93874418982923	8.73013247394416
H	18.70202707646236	5.94920955278508	4.18812828769261
H	18.16174210178157	0.53084276876150	5.38131708092332
C	25.58818001536188	12.58769000017949	8.36217004875372
H	24.50991000095441	12.52163007193476	8.54425999653944
C	25.89372997986481	13.70199003216659	7.36126999875121
H	26.97789999049255	13.86237002277189	7.31791000431982
C	25.37533006677682	13.32638003621827	5.98205001004823
H	24.27990999572583	13.26883002740063	5.97364999969442
C	26.00358990399100	11.99295009481638	5.56659009263717
H	27.08825000495552	12.12485005629982	5.46346002088481
C	25.71243999586178	10.93283962133190	6.64148946441677
H	24.64188994087663	10.70944005984916	6.64814021476019
C	26.40031526642926	9.57505181418780	6.37056839957208
H	27.47447388556579	9.72758734647829	6.13774485006810
H	26.34481793704517	8.97951925646376	7.30981720183186

O	25.23784606353038	14.87270370937184	7.84184689563191
O	25.77957861582660	14.39871833348429	5.13396435471512
O	25.46846403407631	11.50507882738453	4.34930521045597
O	26.15855561579782	11.36005686782352	7.91578697069368
O	25.84582169572377	8.84587811235886	5.27868058038956
H	25.55464318172778	14.14110173143379	4.20549464431766
H	24.95596293320198	8.49921757988873	5.51661264616788
C	19.25739000314538	3.71558994394366	13.54451000617291
H	20.33536999366315	3.78280006908652	13.73081000696568
C	18.96503999528761	2.58337000392308	12.56039999332605
H	17.88281999883787	2.40407000170262	12.51415000062672
C	19.47022000135264	2.95813000098886	11.17255999774031
H	20.56565000208785	3.01760999529026	11.16018000248606
C	18.84514999349272	4.29869002322046	10.75849998352354
H	17.76004999726955	4.16953999790560	10.65474000100824
C	19.13531999192686	5.36256002833812	11.82841999648913
H	20.20580000714077	5.58670996836688	11.83615001585684
C	18.34993000175915	6.64921004381299	11.53608994910924
H	17.32957000072168	6.39988000005270	11.22617000380270
H	18.26985000117365	7.24687000101006	12.45040000118256
O	19.66806514666325	1.43732432346875	12.99778634914469
O	19.04561426554150	1.92198330195060	10.29696278967684
O	19.38353434212080	4.77760560742980	9.53848264502472
O	18.69442045292183	4.94812803292156	13.11080720236865
O	18.91391431141718	7.44131889877368	10.49106479372990
H	19.36899813363054	1.22616790459935	13.91712690563175
H	19.30319821328224	2.19935690750130	9.38722044939967
H	19.78962260527596	7.76983015656850	10.78454777546743
C	22.53143997144919	7.83455966193674	10.82294940651823
H	23.59490998705883	8.03634023336431	11.00214974895135
C	22.40688017935958	6.65347002416813	9.85342982621115
H	21.37217994026591	6.30593002211841	9.80962019182629
C	22.88809959228479	7.03836033737640	8.46013007985600
H	23.98287008112041	7.06352994205074	8.44136998966957
C	22.29863028981258	8.38963009498264	8.02989021407731
H	21.22561996175584	8.26468992525085	7.84413986684961
C	22.47558996895345	9.44503005836372	9.12938998886260
H	23.54195001697869	9.65438993572489	9.28017993455242
C	21.77141999656682	10.75757999107040	8.77202999611918
H	20.73224999456436	10.55920999973767	8.48933999622470
H	21.75544999464691	11.40851999882898	9.65153000903772
O	23.23142826876795	5.61217767425158	10.34959543203484
O	22.42615980310682	6.03506848547065	7.55829589205025
O	22.92373576963595	8.88321187479010	6.85049432489160
O	21.86578537549901	9.00854395149470	10.34248105794945
O	22.46473519007883	11.45793716889268	7.72302751705810

H	22.84852470738409	5.33971068610335	11.21809502111776
H	23.16381848189856	5.63743471886399	7.03263043914324
H	22.15552884509144	12.38366357151710	7.74397126911476
C	26.12065999087013	11.86153001622320	13.54481998769413
H	27.20221000135712	11.93311000216070	13.72135999738001
C	25.82281002739597	10.73087990154034	12.55969003862825
H	24.74519997930737	10.55775011618023	12.51119995939878
C	26.32398998296852	11.10342002162460	11.17153001764598
H	27.41912999982059	11.16325999896140	11.15936999621527
C	25.70315000037846	12.44785999851223	10.75981999991504
H	24.61726999745987	12.32050999981918	10.65201000229203
C	25.99711999960133	13.51075999937412	11.83290000001818
H	27.07205999943520	13.74061999982815	11.83836999891183
C	25.21174000019149	14.79465000144953	11.53134999766912
H	24.19115999900672	14.55407999937721	11.21002999838750
H	25.11485999907353	15.39065999984260	12.44426000035767
O	26.47560524059127	9.55329630822721	13.00504895794485
O	25.89007569101964	10.06323978911129	10.28762274005151
O	26.24369337866736	12.94063530076918	9.53817971821639
O	25.55442582846968	13.08963336186375	13.11424947179632
O	25.85722073347452	15.63043490128494	10.59216603289386
H	26.12930836548161	9.34418799628216	13.91040420549266
H	26.13899066019417	10.34483224729775	9.37601680232948
C	22.22384000309800	8.45910002141602	15.97585998734947
C	22.40805999870461	9.64894000391307	15.03087000816921
H	23.45669001149045	9.95827996566298	14.99912999625563
C	21.94670000881934	9.26594997320636	13.63161000824288
H	20.85294000153730	9.23965000355492	13.60640999690111
C	22.53997997143624	7.91098002064191	13.20422997395373
H	23.61551999992476	8.03285998827290	13.02623000662419
C	22.36087006022606	6.85008982918053	14.30277009711425
H	21.29650994796049	6.62870019377300	14.44269988653738
C	23.09769075115464	5.53810093814931	14.00595583602293
H	24.14781331296009	5.75963959586506	13.70704371430479
H	23.12545736370141	4.96465471206850	14.95873890174009
O	21.64915482796307	10.72242172814597	15.58862514410439
O	22.37260152757636	10.31551865585523	12.75451086440759
O	21.90733824833795	7.45579196525156	12.00990843334914
O	22.92916460990028	7.33510413556714	15.50996211660825
O	22.41427879591505	4.80133466397074	12.98338008208622
H	21.84031767897378	11.50251113961474	15.03353122182337
H	22.07517812485186	10.06877339313206	11.84533635468104
H	22.75437557370642	3.88637765045079	12.99176463255926
C	18.65217999968273	4.43251999834257	18.68929999751552
C	19.00101999197140	5.56902000300669	17.72952999834052
H	20.08904999965592	5.71781999838213	17.70012999745750

C	18.51443002347536	5.19684994755146	16.33900003062189
H	17.41944000197117	5.13962000401380	16.32171999230243
C	19.13618999646483	3.84687999997060	15.93549000538627
H	20.22367999996644	3.97440999792734	15.83557999904638
C	18.84339999897753	2.78068999823635	17.00614000258101
H	17.77027000220260	2.54287999924600	17.00843000107416
C	19.58633012317983	1.45156133516565	16.75744536683635
H	20.65371036565775	1.65293111213422	16.53339307450468
H	19.55036393856217	0.87321024993567	17.70962490585238
O	18.35278545276071	6.75477112905729	18.15592674079419
O	18.95313296691927	6.22875315758954	15.45541151583501
O	18.59093322369858	3.36662696709698	14.72139206525038
O	19.26010686789926	3.21564734847017	18.28813740914042
O	19.08525209654038	0.69297297476198	15.66437336468849
H	18.54190307202213	6.81601357937027	19.11484895905120
H	18.72792270520372	5.93893251804084	14.53602763019190
H	18.14036256516226	0.50712899390590	15.82213693041977
C	25.50858999339333	12.58265001112049	18.70100001033711
C	25.86082000072370	13.71004999874761	17.72955999739576
H	26.94982000080770	13.84960999914411	17.70298999918123
C	25.36856998983130	13.33356999605170	16.34137998923984
H	24.27345999857660	13.27450999894130	16.32528000022142
C	26.00181001256181	11.99580002789001	15.93517002778350
H	27.08733000044944	12.12649000226040	15.83735999771585
C	25.70805000151249	10.93637993304553	17.00640992529829
H	24.63535998912542	10.71759004995540	17.00908004234804
C	26.39140436582800	9.57828729022661	16.73390344005848
H	27.44774528394623	9.73672440335565	16.43230879197657
H	26.39816074372498	9.02500260965745	17.69988015968559
O	25.23545980429746	14.91031496603579	18.15381270360053
O	25.77737616388213	14.40623318424907	15.49328477310146
O	25.46530940498506	11.50386079638324	14.71904917817983
O	26.13594486621945	11.37482378652170	18.27633021363596
O	25.78048135007614	8.81595749801479	15.69941322175139
H	25.56979020677710	14.14147159257536	14.56282036602759
H	24.91529412849475	8.47868525975864	16.01922963807055
H	24.42291999964691	12.47615000226403	18.83228999757528
O	25.95667851189636	12.85089796187037	19.99614351830527
H	17.56731000016062	4.32562000031104	18.82683000005668
O	19.11410964870945	4.73660097911231	19.98393924513836
H	20.09169579020069	4.77000276712826	19.93005940281700
H	26.88553790540609	13.14655802057418	19.92949841822117
H	21.16255999859426	8.23139999537273	16.13268000202228
O	22.82712262040723	8.75969611739677	17.22160174664180
H	22.53031290111358	9.67273986489158	17.42382670548356
H	19.20838834210239	5.66778255316333	-0.97777972704091

H	22.87439542642141	8.24856078257460	-4.23300377958964
H	26.01489227512863	12.32483525936376	-1.54272439035014
H	26.01635688542694	14.97136173787506	-0.58471867687088
H	25.72726344040263	15.25502931309007	9.69127439898042
H	25.27547790867297	15.48964543814503	17.36441859190164
H	25.33261230545623	15.53837445563207	7.12948173759244
C	26.34696258875293	5.47949109550629	5.24778428271434
O	24.19409110071892	4.54630786333658	5.86640079100628
H	26.94937374016407	5.59846333275184	4.32823481354128
H	26.88536051791366	4.83760199588328	5.96853632591243
H	26.19926068659726	6.48583679157672	5.68289992807000
H	27.12748731497644	7.79891706836045	1.61986730799632
H	28.52141634994720	6.15930117418888	-0.24696487024233
C	26.94735242262130	6.78275896831719	1.24148949356071
C	27.61652120300704	5.97438930147130	0.33923878870013
N	25.87696653571065	6.03615777556080	1.70674787169658
H	25.25567927212150	6.16604738134149	2.56264058828470
N	26.93837750117519	4.76566560820485	0.28192409147472
C	25.90115296554043	4.82545474296750	1.13072645626799
H	27.28348472115159	3.76939241630635	-0.05070926578215
H	25.19502693938527	4.01127553587999	1.34027491930690
O	27.66496267091317	2.30759178582167	0.04365484691716
H	27.20417948644127	1.69022008803651	-0.55454101677503
H	27.28250668743864	2.08217011744184	0.99587810561411
H	27.57795653836817	3.51235193876235	3.53009566227879
N	25.46021231880350	3.35343751731464	3.94874261045800
C	26.61305100266752	3.03979895384794	3.30749187842459
C	24.48455944926879	2.56593776252856	3.38664493886541
H	23.44818079394677	2.59342881349398	3.74145056849466
N	26.44380283462464	2.08684669693918	2.36377387582090
C	25.09632375802212	1.78647293710334	2.40222790371802
H	24.65212517653745	1.02420390190899	1.74904118862321

Cell-Iβ-OH – AcIm – H_2O – Im (End)

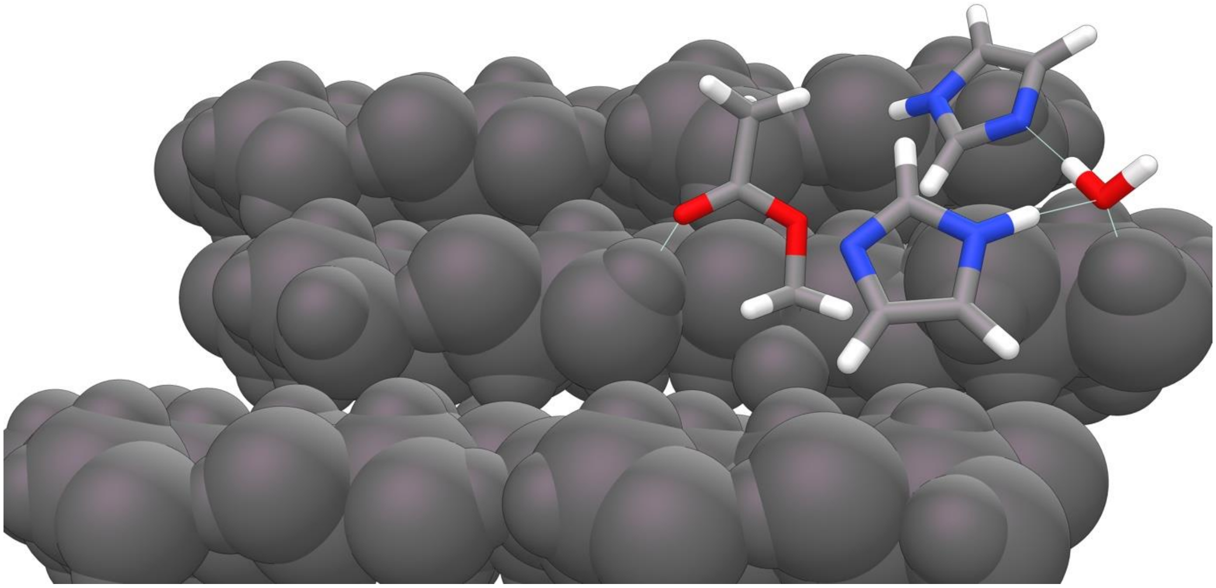

Supplementary Figure 36: Cell-Iβ-OH – AcIm – H_2O – Im (End)

287

Coordinates from ORCA-job orca

C	19.23342990579327	3.71552005581660	3.16692006867584
H	20.30610001912038	3.77884004183824	3.36622985044457
C	18.92928007751590	2.58102981166626	2.18044007165852
H	17.85229000494628	2.39040001279591	2.16355996307818
C	19.35188993361697	2.94280056261876	0.76293985489546
H	20.40923997648535	2.96464956850390	0.63966012932005
C	18.81575008357520	4.30980982870302	0.36278015619508
H	17.73682000110657	4.20885003777952	0.21842993468292
C	19.12620999375781	5.36442980932236	1.44017988303323
H	20.19894996302037	5.58349003536417	1.44841997896397
C	18.34932996286496	6.65546009806793	1.15673000442701
H	17.33060999186582	6.41082998257132	0.83641001799025
H	18.26340000741816	7.24253000717137	2.07684000985306
O	19.63712771526331	1.40873682598117	2.55181655699402
O	18.83041655642230	1.96205229808191	-0.13898432533887
O	19.45094771217011	4.71561089677291	-0.84909498077983
O	18.68423913058081	4.95030568833496	2.73279015870409
O	18.92685966568438	7.38297346959297	0.07034983822502
H	19.40267690027322	1.18177213208103	3.49039335041741
H	19.11949336127032	1.10579750124315	0.23513506980358
H	19.76646531031809	7.78823557873497	0.37615378577974
C	22.51998003471675	7.86374025867716	0.44024860186086
H	23.58635992511193	8.05469005939394	0.61908110104230
C	22.39085037707655	6.71212927378186	-0.56469973504216
H	21.37433986316064	6.31165041116313	-0.57650993641931

C	22.87110978431643	7.07474022331741	-1.94657975830168
H	23.96941004213511	7.07301996787639	-1.95998022225672
C	22.31139001085662	8.41665006001640	-2.37779002206966
H	21.24829002757090	8.28924999448561	-2.61260003836757
C	22.47590004393858	9.46190961717196	-1.26087989739261
H	23.54196993014039	9.66230039092406	-1.09963003843875
C	21.77893000425645	10.76919002386255	-1.61250002345125
H	20.74205000556862	10.57048999134696	-1.90877999871238
H	21.74672999477229	11.40637999836354	-0.72397999875456
O	23.20061477601176	5.64870695889623	-0.03409398042330
O	22.49528656027904	6.10120207931020	-2.91936731301595
O	22.99123316924097	8.90460907519182	-3.53835906034159
O	21.85839018044367	9.01271033618160	-0.04332031221209
O	22.50016895438750	11.47298431775481	-2.61042934405978
H	22.67532216781112	4.82345614979505	-0.00977267938513
H	23.19350399279961	5.40001061566348	-2.83638587353664
H	22.68198404312480	10.81415878951585	-3.31749481454607
C	26.11994998252052	11.86512999644071	3.16892998776323
H	27.20166000173272	11.93419000316685	3.34483999543280
C	25.82213999966248	10.73484000665886	2.18163009337824
H	24.74503001160439	10.55882002912876	2.13195993355198
C	26.32664004822020	11.10873988548541	0.79157000065555
H	27.42219999254137	11.16070001637275	0.78085000570533
C	25.71789998779755	12.45502999047001	0.37544998139047
H	24.63556999689131	12.32599000367830	0.23878999583887
C	26.00105999999654	13.51812999803804	1.44992000075631
H	27.07496000471871	13.74860999695865	1.45841999995183
C	25.20907000122377	14.79783999847204	1.15019999924884
H	24.18815999921156	14.54962999944311	0.83747000306307
H	25.11627000400411	15.39762999961185	2.06125999897316
O	26.47454919728276	9.55045457977170	2.67337021521715
O	25.87739270345343	10.18630372092122	-0.21526135396145
O	26.28920161605644	12.92640329241967	-0.84047801503464
O	25.56075582701769	13.08616459357299	2.73546185545524
O	25.86670355256280	15.57821722690799	0.16895690390037
H	26.14676281954413	9.37771024496110	3.60401683921016
H	26.52556740579392	9.45816697950736	-0.31690029479939
C	22.30687998094199	8.49990974202547	5.65848954947426
H	21.24552001726222	8.28272017760639	5.82869031778688
C	22.43701999202531	9.66219999965642	4.66995090970723
H	23.47526004677612	9.99797001919261	4.62245919641432
C	21.95800998198269	9.27431998432528	3.27108002609608
H	20.86353000869828	9.24016998660549	3.25126000022042
C	22.54910998908799	7.92624997598323	2.84619001830532
H	23.62281000625070	8.03992000000931	2.65823000002674
C	22.35336996050431	6.91792005755571	3.99581998187115

H	21.28751003547974	6.69818990650602	4.13095002933218
C	22.98914703918965	5.56888239217469	3.64057881034888
H	22.73990036335495	4.80021249983191	4.39624511961761
H	22.66023393321234	5.24145982872075	2.63819463660998
O	21.63071763151827	10.73592022399463	5.13429588952891
O	22.39560510705537	10.30491255227403	2.37861697321546
O	21.91560340966361	7.46470276078885	1.64427685996967
O	22.98813073565977	7.37488001957905	5.16417942322567
O	24.42197508941252	5.69963484211439	3.55274550094217
H	21.98334150189095	10.97215847170393	6.02645463727371
H	22.04640797673308	10.07702594775571	1.48485074478947
C	25.13465163085728	5.44934656520327	4.68339191378339
C	18.72707998210483	4.43237004458074	8.35972901075201
H	17.64541000661116	4.36083001341310	8.53470001251985
C	19.02502911938215	5.56450344916005	7.37388381911977
H	20.10251085254010	5.73760661586677	7.32358621121463
C	18.52408997464431	5.19349005060464	5.98448000533947
H	17.42866000075522	5.13587000805010	5.97016000591761
C	19.14032003232885	3.84879885659703	5.56906987221099
H	20.22677999016373	3.97463016215117	5.45950011843904
C	18.84933997585640	2.78409998377493	6.64009007619715
H	17.77499000690496	2.55239001000995	6.64633996345780
C	19.58797731313323	1.45320484429908	6.36297438422813
H	20.65863275233458	1.65762369551815	6.16237639480722
H	19.53506336427481	0.83403522081777	7.28808665591565
O	18.35909426000631	6.73616886557881	7.81322450434797
O	18.95562788405851	6.23001888838831	5.10130353706650
O	18.57813071038516	3.37180968739722	4.35364286544723
O	19.29004605815965	3.19674790648362	7.91606967989263
O	19.09708803300872	0.72997473256467	5.23803765366777
H	18.66911852727946	6.93462094020351	8.73372176208466
H	18.70376853751027	5.95125232047424	4.18732386585379
H	18.14567400076533	0.55917201655270	5.37356271263867
C	25.58817999834495	12.58769000462600	8.36217000231092
H	24.50991000081711	12.52163000104797	8.54426000189354
C	25.89373000219594	13.70199000092648	7.36127000106864
H	26.97789999970490	13.86237000316892	7.31790999858738
C	25.37533000159206	13.32638000365206	5.98205000366870
H	24.27991000224220	13.26883000144842	5.97364999828177
C	26.00359001257601	11.99294998897080	5.56658999177316
H	27.08825000446965	12.12484999915421	5.46345999486215
C	25.71243996556630	10.93284002025850	6.64149001636904
H	24.64188999708670	10.70944003031940	6.64814000410009
C	26.40820942268824	9.57828210425794	6.37499075057216
H	27.47915552343355	9.73772738310467	6.13331510958153
H	26.36208219659964	8.98459884388673	7.31470991558535

O	25.23784638626250	14.87216425588077	7.84123611040407
O	25.78014994036765	14.39870081720146	5.13543373044410
O	25.46997758531091	11.50249010654000	4.34895627419020
O	26.15825086004507	11.36031277848322	7.91479483599917
O	25.85131144666790	8.83874613369192	5.28836806503211
H	25.55058542043713	14.14703282892217	4.20700291884877
H	24.96296509367090	8.49467437896843	5.53770491372596
C	19.25738999226130	3.71558999866564	13.54451000183482
H	20.33537000336835	3.78279999714513	13.73081000104960
C	18.96504000042939	2.58336999855595	12.56039999962010
H	17.88281999886686	2.40407000180722	12.51415000067014
C	19.47022000159738	2.95813000048935	11.17255999823038
H	20.56565000194378	3.01761000060181	11.16018000271094
C	18.84514999960300	4.29868999598292	10.75849999851138
H	17.76004999777263	4.16953999760114	10.65474000078694
C	19.13531999813051	5.36255999523651	11.82842000558820
H	20.20579999748747	5.58671001506458	11.83614999428537
C	18.34993000212374	6.64921101182660	11.53608899046269
H	17.32957000070990	6.39987999954108	11.22616999802382
H	18.26985000129850	7.24687000106627	12.45040000043449
O	19.66826584409901	1.43724423584963	12.99743067586940
O	19.04576207912321	1.92154720851624	10.29852322289138
O	19.38376790660148	4.77743859596210	9.53837128062334
O	18.69473435361418	4.94786713003137	13.11055189128644
O	18.91480029953945	7.44109584053082	10.49134662303062
H	19.36925365736407	1.22474767104910	13.91669704481723
H	19.30298051289936	2.19520645874163	9.38764410585659
H	19.78904305643523	7.77258819416209	10.78589250518264
C	22.53144000402400	7.83455491824604	10.82295407958829
H	23.59491000634424	8.03634305207888	11.00214694037701
C	22.40687992231242	6.65347106925353	9.85342703197993
H	21.37218001275687	6.30592997801490	9.80962196431274
C	22.88810015330572	7.03836085369160	8.46013093251259
H	23.98286998749526	7.06352999103574	8.44137002834533
C	22.29862997486712	8.38962998653607	8.02988998646602
H	21.22561998885888	8.26469000169136	7.84414002236773
C	22.47558999493896	9.44503000563059	9.12938999387435
H	23.54194999992266	9.65438999395231	9.28017999735527
C	21.77142000150595	10.75757999752186	8.77203000096261
H	20.73224999964789	10.55921000025998	8.48934000129317
H	21.75545000010931	11.40851999820622	9.65152999826940
O	23.23132031244892	5.61311512784901	10.35095330731379
O	22.44992186372420	6.01853764208945	7.56483376775705
O	22.92546859953247	8.88112884667948	6.84993913836285
O	21.86608401651490	9.00867243101173	10.34190247403860
O	22.46596007949617	11.45918860845355	7.72328699012131

H	22.85063679381333	5.34205555064130	11.22123415600828
H	23.15154917677950	5.83968861717006	6.89781719877389
H	22.15339706275967	12.38395685931219	7.74102992942740
C	26.12066000149947	11.86153000554539	13.54481999302616
H	27.20221000139511	11.93311000211069	13.72135999739708
C	25.82281000629613	10.73087997574251	12.55969001749759
H	24.74520000037354	10.55775003684803	12.51119998050368
C	26.32398998829185	11.10341998971992	11.17152999099446
H	27.41913000003065	11.16326000488295	11.15937000176897
C	25.70315000049120	12.44785999881057	10.75982000003082
H	24.61726999747319	12.32051000008592	10.65201000234920
C	25.99711999959301	13.51075999955584	11.83290000020695
H	27.07205999937802	13.74061999993513	11.83836999901597
C	25.21174000019099	14.79465000150797	11.53134999777736
H	24.19115999902569	14.55407999936605	11.21002999839859
H	25.11485999900991	15.39065999985169	12.44426000038594
O	26.47382293482644	9.55246164943646	13.00468775193728
O	25.89159683484424	10.06121174232361	10.28958013050970
O	26.24417869891995	12.93898642244831	9.53741000541681
O	25.55428785012344	13.08912800261906	13.11348883248172
O	25.85713909148781	15.63114577235812	10.59312129572190
H	26.12578820652933	9.34312087386878	13.90941678171594
H	26.14703656898579	10.33674165148548	9.37847386886949
C	22.22383999244835	8.45909999103262	15.97586000811766
C	22.40806004093321	9.64894000456110	15.03086994459908
H	23.45668996458746	9.95828001183833	14.99913003926507
C	21.94670001366257	9.26594998396101	13.63161001329643
H	20.85293999610982	9.23964999830761	13.60640999682376
C	22.53997994956023	7.91098000390067	13.20422998775488
H	23.61552001040401	8.03286000425634	13.02623001190781
C	22.36087005513351	6.85008996518861	14.30276999541936
H	21.29650998193089	6.62870001201452	14.44270000854264
C	23.09587533009583	5.53615352480812	14.00859243124458
H	24.14795398429074	5.75600507306910	13.71539914397582
H	23.11616753145258	4.96278699114433	14.96098853064868
O	21.64842595728592	10.72280574248214	15.58694287914217
O	22.37319568294449	10.31511952425751	12.75523054662965
O	21.90749746277364	7.45643307174490	12.00919393096590
O	22.92846881112566	7.33492444217939	15.50984795874243
O	22.41677171005268	4.80020651935541	12.98214079144119
H	21.82329700442178	11.49797131730912	15.01918989589333
H	22.06451129841491	10.07588587047418	11.84828417758004
H	22.75334496202499	3.88387365816667	12.99321982711395
C	18.65217999980699	4.43251999830421	18.68929999755835
C	19.00102000271740	5.56901999783397	17.72952999801077
H	20.08904999981069	5.71781999842624	17.70012999758005

C	18.51443000102616	5.19684999993673	16.33899999597378
H	17.41944000187578	5.13961999912075	16.32171999817556
C	19.13619000165370	3.84687999996690	15.93549000007275
H	20.22368000001901	3.97440999804877	15.83557999900113
C	18.84339999892527	2.78069000364497	17.00613999737462
H	17.77027000226942	2.54287999922728	17.00843000102008
C	19.58440344503737	1.45123060256903	16.75474238431574
H	20.65073154885100	1.65175612197672	16.52564878193760
H	19.55171353453650	0.87231048738491	17.70645314439993
O	18.35469180210810	6.75514596890851	18.15728481482435
O	18.95191972818845	6.22995802305859	15.45673214678175
O	18.59095750176002	3.36747589694712	14.72121052501524
O	19.25970954346696	3.21553570772480	18.28813964149008
O	19.07730390740672	0.69443916473068	15.66314553190223
H	18.53080414621020	6.80561662743587	19.11937600993377
H	18.72322726897419	5.94396599568615	14.53715933020619
H	18.12746495177254	0.52955849726742	15.81555331978949
C	25.50858999391277	12.58265000575117	18.70100000532615
C	25.86082001125935	13.71005001504364	17.72955999167846
H	26.94981999557719	13.84960999393041	17.70299000441333
C	25.36856996891726	13.33357000101180	16.34137998927563
H	24.27346000372501	13.27450999853871	16.32528000584555
C	26.00181003390296	11.99579998566079	15.93516998088129
H	27.08732999541715	12.12649001828384	15.83736001368198
C	25.70805000521221	10.93637986956819	17.00640992885822
H	24.63535998966909	10.71759010324106	17.00908008019018
C	26.38760677264196	9.57767170764910	16.72937214642899
H	27.44105836329043	9.73486018344908	16.41730535429335
H	26.40199510777984	9.02414430755896	17.69486610860704
O	25.23603277279151	14.91041692137618	18.15325545610291
O	25.77837713831248	14.40652968412546	15.49454280170184
O	25.46536748201170	11.50490784403888	14.71848224649749
O	26.13579428122393	11.37457722909948	18.27592965823719
O	25.76590629566843	8.81762255218424	15.69979334323698
H	25.56929062585992	14.14570933397251	14.56379049284769
H	24.90222617462867	8.48229523915600	16.02622999929211
H	24.42291999892424	12.47615000154684	18.83228999717820
O	25.95742283185774	12.84983107856565	19.99573508454287
H	17.56731000023096	4.32562000030020	18.82683000008961
O	19.11464297732134	4.73677872473244	19.98361994945076
H	20.09219655382075	4.76933209172779	19.92899401609508
H	26.89016936673602	13.13333672052061	19.92866524363378
H	21.16255999820471	8.23139999508019	16.13268000260268
O	22.82834566114886	8.75760828332650	17.22125443110338
H	22.53234764480734	9.66953310553331	17.42793817023021
H	19.22283373289981	5.66525316145347	-0.97526762229389

H	22.94323600061750	8.16902534624617	-4.18344461885848
H	26.03714663411298	12.26941833447781	-1.52057557616530
H	26.00862636848129	14.97148654143301	-0.58956645234824
H	25.72577282313912	15.25831149922289	9.69181440822767
H	25.28014580896242	15.49109718737567	17.36506235028016
H	25.32786774883598	15.53756693442031	7.12795587911572
C	26.60955074658484	5.64434828489216	4.44099293537296
O	24.63711229800164	5.14821516839878	5.75632657664710
H	26.88280402535447	5.42954534145212	3.39191079833894
H	27.18521344156823	5.01016934486585	5.13842918879917
H	26.83986680859145	6.70840504269218	4.65946414678129
H	28.62765578456714	8.37942306077858	0.12523847678784
H	27.96565313897482	6.75102543825635	-2.06458891024776
C	27.78080023933255	7.70524287851744	-0.04372059905595
C	27.44594036621427	6.87548739629682	-1.10715283921057
N	26.82203626180045	7.46742130142296	0.92998674777279
H	26.66887799889844	8.10189950410121	1.75483584155212
N	26.30541635613572	6.16399657643973	-0.79565426462246
C	25.94896231273047	6.54837349636450	0.43724699250733
H	25.35797479684978	5.17888622759730	-1.75784535324619
H	25.06499657467544	6.18440914443461	0.97474837546087
O	24.76917863465869	4.54619325408511	-2.32268602074987
H	25.34989225896384	4.25050701025461	-3.04886018153570
H	24.48227577922940	3.38238839656812	-0.97879822786462
H	25.79074305825467	3.57219818528431	1.23223387833048
N	24.00316264082944	2.77941036641569	2.16079014603440
C	24.77990295614335	3.15365578172537	1.14681160367820
C	22.82396745793486	2.35882536827251	1.57815216723670
H	21.98159604077398	1.96483939225566	2.16477472159271
N	24.16814724277663	2.97947117351937	-0.06654075574850
C	22.89819652618405	2.49072221672567	0.19119095166804
H	22.19297372220192	2.24875414367204	-0.61239953467602
